# The limitless endophytes: their role as antifungal agents against top priority pathogens

**DOI:** 10.1186/s12934-024-02411-3

**Published:** 2024-05-31

**Authors:** Ashaimaa Y. Moussa

**Affiliations:** Department of Pharmacognosy, Faculty of Pharmacy, Ain-Shams University, African Union Organization Street, Abbassia, Cairo, 11566 Egypt

**Keywords:** Antifungal, Multi-resistant fungi, Coculture, Fungal biofilm, Endophytes

## Abstract

Multi resistant fungi are on the rise, and our arsenal compounds are limited to few choices in the market such as polyenes, pyrimidine analogs, azoles, allylamines, and echinocandins. Although each of these drugs featured a unique mechanism, antifungal resistant strains did emerge and continued to arise against them worldwide. Moreover, the genetic variation between fungi and their host humans is small, which leads to significant challenges in new antifungal drug discovery. Endophytes are still an underexplored source of bioactive secondary metabolites. Many studies were conducted to isolate and screen endophytic pure compounds with efficacy against resistant yeasts and fungi; especially, *Candida albicans, C. auris, Cryptococcus neoformans and Aspergillus fumigatus,* which encouraged writing this review to critically analyze the chemical nature, potency, and fungal source of the isolated endophytic compounds as well as their novelty features and SAR when possible. Herein, we report a comprehensive list of around 320 assayed antifungal compounds against *Candida albicans, C. auris, Cryptococcus neoformans and Aspergillus fumigatus* in the period 1980–2024, the majority of which were isolated from fungi of orders Eurotiales and Hypocreales associated with terrestrial plants, probably due to the ease of laboratory cultivation of these strains. 46% of the reviewed compounds were active against *C. albicans,* 23% against *C. neoformans,* 29% against *A. fumigatus* and only 2% against *C. auris*. Coculturing was proved to be an effective technique to induce cryptic metabolites absent in other axenic cultures or host extract cultures, with Irperide as the most promising compounds MIC value 1 μg/mL. *C. auris* was susceptible to only persephacin and rubiginosin C. The latter showed potent inhibition against this recalcitrant strain in a non-fungicide way, which unveils the potential of fungal biofilm inhibition. Further development of culturing techniques and activation of silent metabolic pathways would be favorable to inspire the search for novel bioactive antifungals.

**Graphic abstract:**

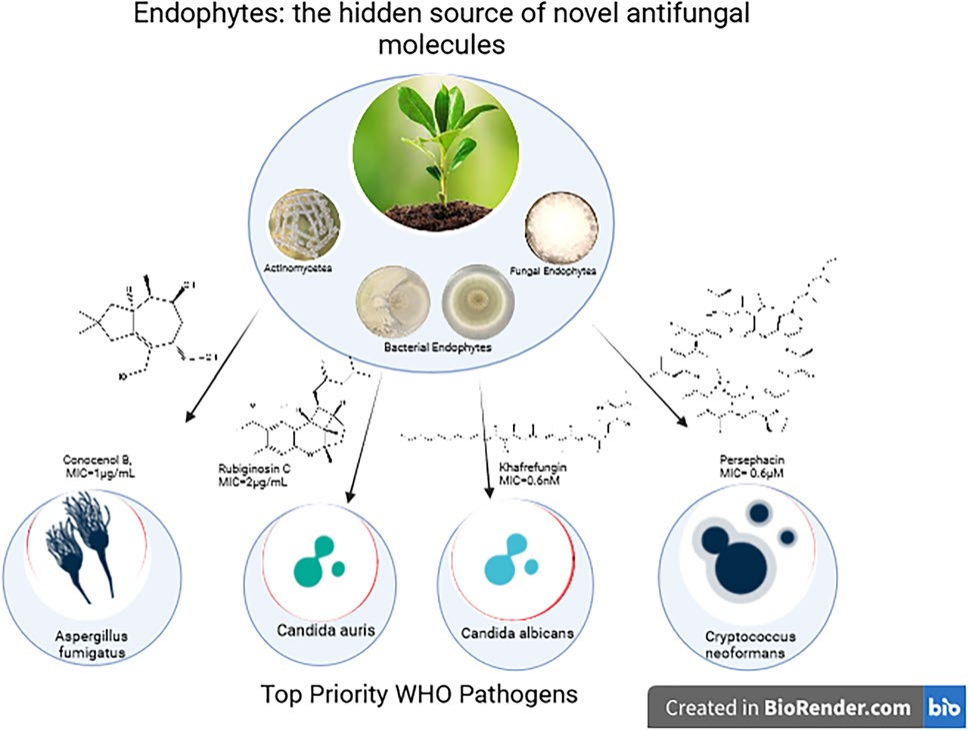

## Introduction

Antifungal resistance was underestimated for a long period of time. The most pronounced cases were seen in patients with cancer therapy, organ, or bone marrow transplantation. Currently, a huge deficiency is encountered in the market regarding the antifungal drugs effective against systemic and local infections; particularly, with the emerging multi-resistant fungal strains [1]. The WHO report in 2023 listed three fungal priority pathogens, *Candida auris, Aspergillus fumigatus and Cryptococcus neoformans* and urged the critical importance of developing new effective drugs against them [2].

Endophytes are the microbial community associated with plants with no significant harm, which was known to provide the plant with marked natural products diversity as well as disease, insects, nematodes defiance [3, 4]. These largely untapped and sustainable resources of natural products have revolutionized the field of drug discovery since it provided novel molecular skeletons in mass yield [5, 6]. It is estimated that endophytes repository of bioactive molecules (80%), particularly the novel ones, could exceed those reported from soil microorganisms; hence, exploring endophytes is an outstanding approach to fight antifungal resistance [6].

Despite the ubiquitousness of *Aspergillus* spores everywhere and the fact that most people can inhale them without hazard, those with severe respiratory infections, hospitalized or under chemotherapeutic regimens can be extremely vulnerable to them. Aspergillosis is life threatening in patients with underlying diseases or immunocompromised patients and is the most common missed diagnosis in intensive care units [7]. With the development of antifungal resistance, *A. fumigatus* became on the watch list in the CDC antibiotic resistant threats 2023; especially, its azole resistant strains whose infection is 33% less likely to be treated than other *Aspergillus* strains [8]. Azole resistance can be acquired from the environment without prior exposure to azole fungicides triazole, voriconazole and itraconazole are antifungal agents that remained in the market for a long time effective, cheap and available yet the emergence of resistance has given the problem new dimensions and demanded the discovery of potent alternatives [9].

Another fungi on the list was *C. neoformans,* representing an annual 1 million infections, *and* commenced with inhaling the fungal air-borne spores and progressed to pneumonia and even CNS meningitis, a cryptococcosis scenario that was commonly encountered in immunocompromised patients with organ transplantation, cancer or HIV [10]. With only a few limited choices of antifungal treatments like fluconazole., amphotericin B or 5-fluorocytosine whose costs, toxicity and cost deter their prescription in the first place let alone azole resistance, *Cryptococcus* sp. are largely left untreated with a huge health hazard [11]. *C. auris* emerged recently as a major infection in intensive care units (ICUs) in reports in India, Kuwait and Spain with average 25 days stay patients. Even though *C. auris* isolates appeared to colonize indwelling devise and catheters, they also infected skin and different body sites [12]. This review aims to cover the antifungal activity exerted by endophytic compounds and extracts against three of the WHO top priority pathogens, *A. fumigatus, C. neoformans and Candida species C. auris* and *C. albicans*. Details about the endophyte source, collection, culture, compounds chemistry and biosynthesis, SAR and antifungal properties will be discussed and analyzed. *Aspergillus* diseases were controlled by commonly prescribed azole antifungal agents until recently when azole resistant *A. fumigatus* emerged as a worldwide health threat. Previously effective medications fell short of dealing with this antifungal resistance, which necessitated antifungal drug discovery research. Natural products with MIC values < 10 μg/mL are considered potent and should be given due care to progress with their in-vivo and clinical studies. MIC values between 10 and 100 μg/mL are moderately active and may be further promoted if suitable medicinal chemistry modifications can enhance their efficacy [13].

## Methodology

Papers with reported bioactivity against any of these pathogenic strains, *A. fumigatus, C. neoformans, C. albicans or C. auris* were included. Endophytes of either fungi, actinomycetes or bacteria were included. The literature search period started from 1980 and extended to 2024, and all types of publications, original articles, reviews or reports and commentaries were included. Negative results of antimicrobial assays against any of the strains of interest were listed here and analyzed to help direct future research to study promising compounds only. Boolean search operators like and, or, not, near, * were exploited to narrow down the search items for the best fit of our keywords. Search engines like Web of Science, Reaxys, Scopus, Google Scholar, Pubmed and Science direct were utilized. Phrase and keywords used were *C. auris* antifungal (1099 results), *C. albicans* antifungal (18,297 results), *A. fumigatus* antifungal endophytes (4958 results), *Cryptococcus neoformans* antifungal endophytes (24 results), bioactive compounds endophytes *C. auris* (307 results), bioactive compounds endophytes *C. albicans* (12,900 results), bioactive compounds endophytes *A. fumigatus* (8320 results), bioactive compounds endophytes *Cryptococcus neoformans* (2750 results). The total number of initial search results was 48,655, which was narrowed further to 110 articles. Results were refined to only the English language articles in top peer reviewed journals, and highly cited articles were prioritized. All articles filtration criteria were conducted according to the Web of Science (WOS) core collection selection.

### A. Terrestrial plant-endophytes

#### I. Bacterial endophytes with activity against selected pathogens

The moderately active antifungal agent toxoflavin was isolated from *Lycoris aurea* bacterial endophyte and optimized in large scale fermentation to yield more than 1300 mg/litre; additionally, the azole resistant human pathogen *A. fumigatus and C. neoformans* were susceptible to toxoflavin [13]. The bacterial endophyte *Bacillus velezensis* LDO2 isolated from peanut was active against *A. flavus* mycelial growth 80.77%, and this was related to the fungicidal compounds fengycin, bacilysin, and surfactin indicated in the UPLC-MS analysis [14].

Three *Bacillus* strains, *B. cereus* (LBL6), *B. thuringiensis* (SBL3) and *B. anthracis* (SBL4) were isolated from *Berberis lyceum,* and their ethyl acetate extract displayed activity against *A. niger and A. flavus* [15]. Bacterial endophytes colonizing the same biological niche with fungi possibly produce metabolites to antagonize and hinder their growth. This was seen in many cases as in cannabis seedling endophytes, which possessed antibiotic activity against its *Aspergillus* pathogen as well as *Alternaria, Penicillium, and Fusarium sp.* Isolation of the bioactive metabolites is highly urged here to progress into discovery and optimization of potential antifungal molecules [16]. In the same vein, *B. velezensis* isolated from grapevine were protective against other grapevine-endophytic fungi including *Aspergillus* spp. Evidently, several lytic enzymes were revealed using molecular genome mining tools as proteases, cellulases and chitinases as well as functional genes encoding macrolactin, fengycin, iturins, difficidin, and mycosubtilin secondary metabolites, which were shown by PCR analysis [17].

#### II. Fungal endophytes with activity against selected pathogens

##### 1. Terpenes

**Fig. 1:**
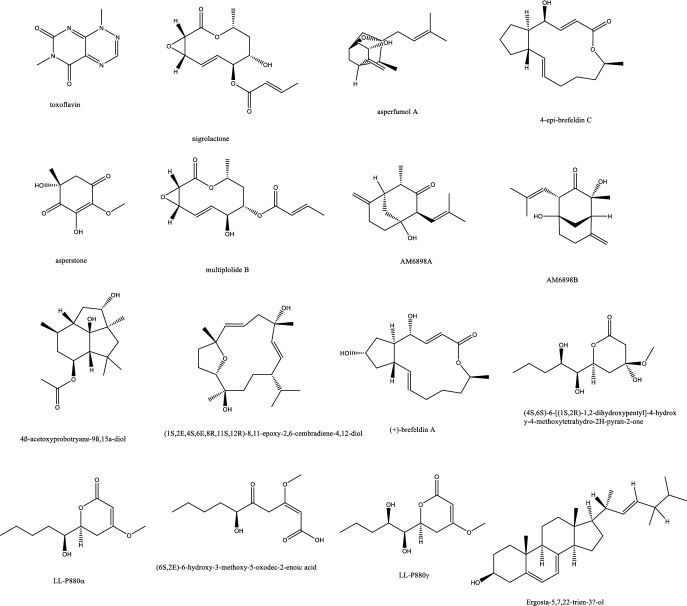
Endophytic terpene compounds with antifungal potential activity

On the other side, monoterpenes from the endophytic *Pestalotiopsis foedan* were only weakly active with MIC value of 50 μg/mL [18]. *Nicotiana tabacum* endophytes produced several molecules, a fumagillol derivative, a 10-membered lactone, a cyclohexanones together with sesquiterpenes and cembrdiene diterpenes with promising antifungal affect against *A. fumigatus* and MICs ≤ 8 μg/mL [19] (Table 1). (S, Z)-phenguignardic acid methyl ester, a meroterpene of the guignardianone type was isolated from the endophyte *Phyllosticta sp* J13-2-12Y and manifested a potent effect against *C. albicans.* These meroterpenes are rare in nature and comprised of an amino acid derived benzylidene dioxolanone while the guignardone type compounds formed of a monoterpene linked to a C-7 carbon unit were devoid of considerable activity [20]. Monoterpenes of the P*estalotiopsis* endophytic isolate from *Dendrobium officinale* of Yandang Mountain in China possessed significant antifungal effect against *C. albicans, C. neoformans, T. rubrum, and A. fumigatus* [21]. The triterpene glycoside enfumafungin was isolated from some type of *Kabatina* species inhabiting the leaves of *Juniperus communis* with an activity towards *A. fumigatus* resembling the approved fungicide caspofungin acetate and its precursor pneumocandin B_0._ *In-vivo* studies in rats challenged with *C. albicans* to cause candidemia recorded moderate activity with ED_90_ of 90 mg/Kg with morphological alternations suggesting cell wall targeting particularly glucan synthase [22] (Fig. 1). Further chemical modifications and bioavailability studies led to the development of ibrexafungerp with better pharmacokinetics than enfumafungin [23, 24]. Another member of this class of metabolite is arundifungin, which was isolated from *Arthrinium arundinis* and showed glucan synthase inhibitory activity comparable to echinocandin L-733560 and papulacandins, yet the activity was specific to *A. fumigatus and C. albicans* and not *Cryptococcus*. This was rationalized to be due to the presence of 1,6*-β*-glucan or other non-1, 3-*β-d*-glucan components in its cell wall [25].

**Table 1:** Endophytic antifungal natural products with their potential activity against *Candida albicans, C. auris, Cryptococcus neoformans and Aspergillus fumigatus*

No	Extract	Chemical class	Plant source	Endophytes	Activity	References
1	Am6898a	Terpene	*Nicotiana tabacum*	*Aspergillus fumigatus*	Inactive	[19]
2	Asperfumol A	Terpene	*Nicotiana tabacum*	*Aspergillus fumigatus*	Active against* Nigrospora *sp (1 μg/mL),* Phomopsis *sp. (16 μg/mL),* Alternaria *sp. (4 μg/mL),* P. janthinellum *(32 μg/mL)	[19]
3	Am6898b	Terpene	*Nicotiana tabacum*	*Aspergillus fumigatus*	In active	[19]
4	Asperstone	Quinone (polyketide)	*Nicotiana tabacum*	*Aspergillus fumigatus*	Active against* Nigrospora *sp. (1 μg/mL),* Phomopsis *sp. (4 μg/mL),* Alternaria *sp. (64 μg/mL)	[19]
5	Nigrolactone	NA	*Nicotiana tabacum*	Coculture of* Nigrospora *sp. and *Stagonosporopsis *sp.	Against *Aspergillus fumigatus* with MIC 16 μg/mL, active against *Nigrospora* sp. (1 μg/mL), *Phomopsis* sp. (8 μg/mL), *Alternaria* sp. (16 μg/mL), *P. janthinellum* (16 μg/mL)	[19]
6	Multiplolide B	Lactones	*Nicotiana tabacum*	Coculture of *Nigrospora* sp. and *Stagonosporopsis* sp.	Active against *Nigrospora* sp. (1 μg/mL), *Phomopsis sp*. (8 μg/mL)	[19]
7	4β-acetoxyprobotryane-9β, 15α-diol	Bicyclo octane ring	*Nicotiana tabacum*	Coculture of *Nigrospora* sp. and *Stagonosporopsis* sp.	*A. fumigatus* with MIC **2 μg/mL**, against *Nigrospora* sp. with MICs of 1 μg/mL	[19]
8	4-epi-brefeldin C	Macrolide	*Nicotiana tabacum*	*Penicillium janthinellum*	Antifeedant effect with deterrence indices of 21–100%	[19]
9	Brefeldin A	Lactones	*Nicotiana tabacum*	*Penicillium janthinellum*	*NA*	[19]
10	(1S, 2E, 4S, 6R, 7E, 12S)-2, 7-cembradiene-4, 6, 12-triol	Diterpene	*Nicotiana tabacum*	*Penicillium janthinellum*	Against *Nigrospora* sp. with MICs of 1 μg/mL, against *Phomopsis* sp. with MICs of 4, 2 μg/mL. against *A. fumigatus* < **8 μg/mL**	[19]
11	(1S, 2E, 4S, 6E, 8R, 11S, 12R)-8, 11-epoxy-2, 6-cembradiene-4, 12-diol	Diterpene	*Nicotiana tabacum*	*Penicillium janthinellum*	*NA*	[19]
12	Aspergillethers A	Biaryl ether	*Pulicaria crispa Forssk*	*Aspergillus versicolor*	Towards *C. albicans* and *Geotrichium candidum* compared to clotrimazole	[73]
13	Aspergillethers B	Biaryl ether	*Pulicaria crispa Forssk*	*Aspergillus versicolor*	Towards *C. albicans* and *Geotrichium candidum* compared to clotrimazole	[73]
14	22E,24R)-stigmasta-5,7,22-trien-3-Β-ol	Steroids	*Pulicaria crispa Forssk*	*Aspergillus versicolor*	NA	[73]
15	Stigmasta-4,6,8(14),22-tetraen-3-one	Steroids	*Pulicaria crispa Forssk*	*Aspergillus versicolor*	NA	[73]
16	Orcinol	Polyketide	*Pulicaria crispa Forssk*	*Aspergillus versicolor*	NA	[73]
17	Butyrolactones I	Furanones	*Pulicaria crispa Forssk*	*Aspergillus versicolor*	NA	[73]
18	Butyrolactones VI	Furanones	*Pulicaria crispa Forssk*	*Aspergillus versicolor*	NA	[73]
19	Toxoflavin	Alkaloids	*Lycoris aurea*	*Burkholderia gladioli*	*A. fumigatus* and *C. albicans*, *C. neoformans*, and the model filamentous fungus *A. nidulans*. Effective against the azole antifungal-resistant mutants of A. fumigatus MIC = 64 μg/mL	[13]
20	Occidofungin	Peptide	Soybean plant	Burkholderia sp. MS455	Inhibited *A. flavus* growth	[50]
21	Aspertubin A	Globoscinic acid derivatives (lactones)	NA	*Aspergillus tubingensis* S1120 coculture with Red Ginseng	Against *A. tubingensis* with MIC values at** 8 μg/mL** less active against *P. herbarum*	[113]
22	Panaxytriol	Fatty alcohol	NA	*Aspergillus tubingensis* S1120 coculture with Red Ginseng	Against *A. tubingensis* with MIC values at** 8 μg/mL**. less active against *P. herbarum*	[113]
23	Carviolin	Anthraquinone	NA	*Aspergillus tubingensis* S1120 coculture with Red Ginseng	Moderate activity against *A. tubingensis*	[113]
24	Asperic acid		NA	*Aspergillus tubingensis* S1120 coculture with Red Ginseng	Moderate activity against *A. tubingensis*	[113]
25	Asperazine	Pyrrolindole alkaloid	NA	*Aspergillus tubingensis* S1120 coculture with Red Ginseng	Moderate activity against *A. tubingensis*	[113]
26	Irperide	Butenolide (lactones)	Unknown plant	Co-culture of endophyte *Irpex lacteus* and pathogenic *Nigrospora oryzae*	Against *Aspergillus fumigatus*, with MIC values of **1 μg/mL**	[114]
27	( +)-(3S,6S,7R)-tremulene-6,11,12-triol	Sesquiterpene	Unknown plant	Co-culture of endophyte *Irpex lacteus* and pathogenic *Nigrospora oryzae*	Inactive	[114]
28	Lactedine	Sesquiterpene	Unknown plant	Co-culture of endophyte *Irpex lacteus* and pathogenic *Nigrospora oryzae*	Inactive	[114]
29	Nigirpexin C	Azaphilone	Unknown plant	Co-culture of endophyte *Irpex lacteus* and pathogenic *Nigrospora oryzae*	Against *Aspergillus fumigatus*, with MIC values of **2 μg/mL**	[114]
30	Tremulenediol A	Sesquiterpene	Unknown plant	Co-culture of endophyte *Irpex lacteus* and pathogenic *Nigrospora oryzae*	Inactive	[114]
31	Conocenol B	Tremulane sesquiterpene	Unknown plant	Co-culture of endophyte *Irpex lacteus* and pathogenic *Nigrospora oryzae*	Against *Aspergillus fumigatus*, with MIC values of 1 μg/mL	[114]
32	Nystatin	Polyene macrolide	Unknown plant	Co-culture of endophyte *Irpex lacteus* and pathogenic *Nigrospora oryzae*	Inactive	[114]
33	Quinomycin A	Peptide	Fenghuang Mountain nine medicinal plants	*Streptomyces* sp. YHLB-L-2	Active against *Aspergillus fumigatus*, *Cryptococcus neoformans* as well as strains *Aspergillus fumigatus* #176 and #339 (**MIC 16, 4, 16 and 16 µg/mL**)	[93]
34	Paraphaone	Polyketide-terpene hybrid	*Ginkgo biloba*	*Paraphaeosphaeria *sp.	Against *Alternaria alternata* 2 μg/mL, *Beauveria bassiana* 32 μg/mL	[115]
35	Paraphaterpene A	Eremophilane sesquiterpenoid	*Ginkgo biloba*	*Paraphaeosphaeria *sp.	*Beauveria bassiana* 4 μg/mL	[115]
36	Paraconiothin D		*Ginkgo biloba*	*Paraphaeosphaeria *sp.	Inactive	[115]
37	Paraphaterpene B	Eremophilane sesquiterpenoid	*Ginkgo biloba*	*Paraphaeosphaeria *sp.	Inactive	[115]
38	Paraphaterpenes C	Eremophilane sesquiterpenoid	*Ginkgo biloba*	*Paraphaeosphaeria *sp.	Against *Alternaria alternata* 2 μg/mL	[115]
39	Paraphaterpenes D	Eremophilane sesquiterpenoid	*Ginkgo biloba*	*Paraphaeosphaeria *sp.	Against *Alternaria alternata* 2 μg/mL	[115]
40	Alternariol methyl ether	Isocoumarin	*Ginkgo biloba*	*Paraphaeosphaeria *sp.	Against *Alternaria alternata* 2 μg/mL, *Aspergillus fumigatus* 2 μg/mL, *Beauveria bassiana* 1 μg/mL	[115]
41	Penicichrins A	Drimane sesquiterpenoid	*Ziziphus jujuba*	*Penicillium chrysogenum*	*P. chrysogenum* MICs ≤ 2 μg/mL, and moderate effect against *A. alternata* and *A. fumigatus*	[107]
42	Penicichrins B	Drimane sesquiterpenoid	*Ziziphus jujuba*	*Penicillium chrysogenum*	*A. alternata* and *Aspergillus fumigatus* with MICs ≤ **8 μg/mL**	[107]
43	Penicichrins C	Drimane sesquiterpenoid	*Ziziphus jujuba*	*Penicillium chrysogenum*	*A. alternata* and *Aspergillus fumigatus* with MICs ≤ **4 μg/mL**	[107]
44	Monaspurpurone	Tetralone (benzo fused cyclohexanone)	*Ziziphus jujuba*	*Penicillium chrysogenum*	*P. chrysogenum* and *Alternaria alternata* MICs ≤ 16 μg/mL and *Aspergillus fumigatus* with MICs ≤ 2 μg/mL	[107]
45	4-methoxy-3-methylgoniothalamin	Styryl pyrone	*Ziziphus jujuba*	*Penicillium chrysogenum*	*P. chrysogenum*, *Alternaria alternata* and *Aspergillus fumigatus* with MICs ≤ 8 μg/mL	[107]
46	2-hydroxy-l-phenyl-l,4-pentanedione		*Ziziphus jujuba*	*Penicillium chrysogenum*	*Aspergillus fumigatus* with MICs** ≤ 4 μg/mL**	[107]
47	Physcion	Anthraquinone	*Ziziphus jujuba*	*Penicillium chrysogenum*	*P. chrysogenum*, *Alternaria alternata* MICs ≤ 8 μg/mL moderate effect against *Aspergillus fumigatus*	[107]
48	Ergosterol,	Steroids	*Ziziphus jujuba*	*Penicillium chrysogenum*	*P. chrysogenum*, *Alternaria alternata* and *Aspergillus fumigatus* with MICs** ≤ 2 μg/mL**	[107]
49	Ergosta-7,22-dien-3β-ol	Steroids	*Ziziphus jujuba*	*Penicillium chrysogenum*	Active against *P. chrysogenum* MICs ≤ 4 μg/mL	[107]
50	1-phenyl-1,2-ethanediol	Alcohol	*Ziziphus jujuba*	*Penicillium chrysogenum*	Against *P. chrysogenum*, *Alternaria alternate* MICs ≤ 4 μg/mL	[107]
51	Phomopoxides A	Cyclohexenes	*Paeonia delavayi Franch*	*Phomopsis *sp. YE325	Promising α-glucosidase inhibition	[53]
52	Phomopoxide B	Cyclohexenes	*Paeonia delavayi Franch*	*Phomopsis *sp. YE326	Towards *C. albicans* 32 μg/mL and *A. niger* MIC = 64 μg/mL	[53]
53	Phomopoxide C	Cyclohexenes	*Paeonia delavayi Franch*	*Phomopsis *sp. YE327	Promising α-glucosidase inhibition	[53]
54	Phomopoxide D	Cyclohexenes	*Paeonia delavayi Franch*	*Phomopsis *sp. YE328	Towards *C. albicans* and *A. niger* MIC = 64 μg/mL	[53]
55	Phomopoxide E	Cyclohexenes	*Paeonia delavayi Franch*	*Phomopsis *sp. YE329	Promising α-glucosidase inhibition	[53]
56	Phomopoxide F	Cyclohexenes	*Paeonia delavayi Franch*	*Phomopsis *sp. YE330	Promising α-glucosidase inhibition	[53]
57	Phomopoxide G	Cyclohexenes	*Paeonia delavayi Franch*	*Phomopsis *sp. YE331	Towards *C. albicans*	[53]
58	Pyranonigrin A	Lactones	*Malus domestica*	*Aspergillus tubingensis *AN103	Moderate against *F. solani* MLBM227, *A. niger* ATCC 16404, *C. albicans* ATCC 10231, and *A. alternata* MLBM09	[68]
59	Fonsecin	Naphtho-γ-pyrones	*Malus domestica*	*Aspergillus tubingensis *AN103	Potent against *C. albicans* (ATCC 10231)	[68]
60	Tmc 256 A1	Naphtho-γ-pyrones	*Malus domestica*	*Aspergillus tubingensis *AN103	Potent against *F. solani* MLBM227 and *A. niger* ATCC 16404 and moderate against *C. albicans* ATCC 10231 and *A. alternata* MLBM09	[68]
61	Asperazine	Alkaloids	*Malus domestica*	*Aspergillus tubingensis *AN103	Potent against *F. solani* MLBM227 and *A. niger* ATCC 16404 and moderate against *C. albicans* ATCC 10231 and *A. alternata* MLBM09	[68]
62	Botryorhodine A	Lactones	*Bidens pilosa*	*Botryosphaeria rhodina*	Against *A. terreus* was found to be 26.03 μM,	[60]
63	Botryorhodine B	Lactones	*Bidens pilosa*	*Botryosphaeria rhodina*	Against *A. terreus* was found to be 49.7 μM,	[60]
64	Botryorhodine C	Lactones	*Bidens pilosa*	*Botryosphaeria rhodina*	Inactive	[60]
65	Botryorhodine D	Lactones	*Bidens pilosa*	*Botryosphaeria rhodina*	Inactive	[60]
66	Enfumafungin	Triterpene	*Juniperus communis *leaves	Undetermined *Kabatina *species	MICs < **0.5 μg/mL** against *Candida* and *Aspergillus* species. Activity was shown by both invitro and invivo studies	[22]
67	4-dihydroxy-2′, 6-diacetoxy-3′-methoxy-5′-methyl-diphenyl ether	Ethers	*Rehmannia glutinosa*	*Verticillium *sp.	Against *C. albicans* MIC 8 μg/mL and *A. fumigatus* MIC 16 μg/mL	[74]
68	Paecilospirone	Benzofuranes	*Rehmannia glutinosa*	*Verticillium *sp.	Inactive	[74]
69	Α-acetylorcinol	Polyketides	*Rehmannia glutinosa*	*Verticillium *sp.	*A. fumigatus* MIC** 0.25 μg/mL**	[74]
70	2-methoxy-1,8-dimethyl-xanthen-9-one	Polyketides	*Rehmannia glutinosa*	*Verticillium *sp.	Inactive	[74]
71	4-hydroxy-α-lapachone	Polyketides(quinones)	*Rehmannia glutinosa*	*Verticillium *sp.	Inactive	[74]
72	Enalin A	Benzofuranone	*Rehmannia glutinosa*	*Verticillium *sp.	Inactive	[74]
73	3,4-trimethyl-5,7-dihydroxy-2,3-dihydrobenzofuran	Benzofuran	*Rehmannia glutinosa*	*Verticillium *sp.	Inactive	[74]
74	4-Dihydroxy-3,5,6-Trimethyl-Methylbenzoate	Phenol	*Rehmannia glutinosa*	*Verticillium *sp.	Inactive	[74]
75	3-isopropenyl-(Z)-monomethyl maleate		*Rehmannia glutinosa*	*Verticillium *sp.	Inactive	[74]
76	Arundifungin	Steroids	Unknown plant	*Arthrinium arundinis*	In vitro effective against *Candida albicans*-MY1055, *C. albicans*-CLY539, *C. glabrata*-MY1381, *C. parapsilopsis*-MY1010, *C. pseudotropicalis*-MY2099, *C. tropicalis*-MY1124, *C. tropicalis*-MY1012, *C. krusei*-CLY549. Not effective in-vivo up to 50 mg/kg daily dose	[25]
77	Ascosteroside	Lanostane triterpene	Unknown plant	*Arthrinium arundinis*	In-vivo anticandidal effect	[25]
78	Asperfumoid	Alkaloids	*Cynodon dactylon*	*Aspergillus fumigatus *CY018	Inhibit *C. albicans* with MICs of 75.0 μg/mL	[29]
79	Asperfumin	Polyketides	*Cynodon dactylon*	*Aspergillus fumigatus *CY018	Inactive	[29]
80	Monomethylsulochrin	Polyketides	*Cynodon dactylon*	*Aspergillus fumigatus *CY018	Inactive	[29]
81	Fumigaclavine C	Alkaloids	*Cynodon dactylon*	*Aspergillus fumigatus *CY018	Inhibit *C. albicans* with MIC 31.5 μg/mL	[29]
82	Fumitremorgin C	Alkaloids	*Cynodon dactylon*	*Aspergillus fumigatus *CY018	inhibit *C. albicans* with MIC 62.5 μg/mL	[29]
83	Helvolic Acid	Steroids	*Cynodon dactylon*	*Aspergillus fumigatus *CY018	Inhibit *C. albicans* with MIC 31.5 μg/mL	[29]
84	5alpha,8alpha-epidioxy-ergosta-6,22-diene-3beta-ol	Steroids	*Cynodon dactylon*	*Aspergillus fumigatus *CY018	Inactive	[29]
85	Ergosta-4,22-diene-3beta-Ol	Steroids	*Cynodon dactylon*	*Aspergillus fumigatus *CY018	Inactive	[29]
86	Cyclo(Ala-Leu)	Peptides	*Cynodon dactylon*	*Aspergillus fumigatus *CY018	Inactive	[29]
87	Cyclo(Ala-Ile)	Peptides	*Cynodon dactylon*	*Aspergillus fumigatus *CY018	Inactive	[29]
88	Physcion	Anthraquinone	*Cynodon dactylon*	*Aspergillus fumigatus *CY018	Inhibit *C. albicans* with MIC 125 μg/mL	[29]
89	7-amino-4-methylcoumarin	Lactones	Ancient *Ginkgo biloba* L. tree	*Xylaria *sp.* YX-28*	Against *S. aureus*, *E. coli*, *S. typhia*, *S. typhimurium*, *S. enteritidis*, *Aeromonas hydrophila*, *Yersinia* sp., *Vibrio anguillarum*, *Shigella* sp., *Vibrio parahaemolyticus*, *C. albicans*, and *A. niger* (MIC = 4–25 μg/mL)	[47, 48]
90	Lecythomycin	Macrolactone	*Alyxia reinwardtii*	*Lecythophora *sp.	*Aspergillus fumigatus* and *Candida kruzei* at MIC of 62.5–125 μg/mL	[61]
91	Amino-3,4-dihydroxy-2-25-(hydroxymethyl)-14-Oxo-6,12-eicosenoic acid	Fatty acid	*Eugenia bimarginata DC*	*Mycosphaerella *sp.	*C. neoformans* and *C. gattii*, 0.49 to 7.82 mM	[11]
92	Myriocin	Peptide	*Eugenia bimarginata DC*	*Mycosphaerella *sp.	*C. neoformans* and *C. gattii*, 0.48 to 1.95 mM	[11]
93	(4S,6S)-6-[(1S,2R)-1,2-dihydroxypentyl]-4-hydroxy-4-methoxytetrahydro-2H-pyran-2-one	Pyranone	*Dendrobium officinale*	*Pestalotiopsis *sp. DO14	MIC values < 25 μg/mL against *C. albicans*, *C. neoformans*, *T. rubrum*, and *A. fumigatus*	[21]
94	(6S,2E)-6-hydroxy-3-methoxy-5-oxodec-2-enoic acid	Fatty acid	*Dendrobium officinale*	*Pestalotiopsis *sp. DO14	MIC values < 25 μg/mL against *C. albicans*, *C. neoformans*, *T. rubrum*, and *A. fumigatus*	[21]
95	LL-P880γ	Lactones	*Dendrobium officinale*	*Pestalotiopsis *sp. DO14	MIC values < 50 μg/mL against *C. albicans*, *C. neoformans*, *T. rubrum*, and *A. fumigatus*	[21]
96	LL-P880α	Lactones	*Dendrobium officinale*	*Pestalotiopsis *sp. DO14	MIC values < 50 μg/mL against *C. albicans*, *C. neoformans*, *T. rubrum*, and *A. fumigatus*	[21]
97	Ergosta-5,7,22-Trien-3β-Ol	Steroids	*Dendrobium officinale*	*Pestalotiopsis *sp. DO14	Inactive	[21]
98	Cladosporin	Isocoumarin	Unknown plant	*Cladosporium cladosporioides*	Against *Cryptococcus neoformans* (IC 50 value of 17.7 μg/mL)	[62]
99	Mycousfuranine	Usnic acid derivatives(benzofuran)	*Eugenia bimarginata*	*Mycosphaerella *sp.	*C. neoformans* 50.0 μg/mL, *C. gattii* 250.0 μg/mL	[78]
100	Mycousnicdiol	Usnic acid derivatives(benzofuran)	*Eugenia bimarginata*	*Mycosphaerella *sp.	*C. neoformans* 50.0 μg/mL, *C. gattii* 250.0 μg/mL	[78]
101	Simplicildones A	Depsidones	*Hevea brasiliensis*	*Simpilcillium *sp.	Weak against *S. aureus* with equal MIC values of 32 mg/m	[63]
102	Simplicildones B	Depsidones	*Hevea brasiliensis*	*Simpilcillium *sp.	Inactive	[63]
103	Simplicildones C	Depsidones	*Hevea brasiliensis*	*Simpilcillium *sp.	Against *C. neoformans* with equal MIC values of 32 mg/mL	[63]
104	Simplicildones D	Depsidones	*Hevea brasiliensis*	*Simpilcillium *sp.	Inactive	[63]
105	Simplicildones E	Depsidones	*Hevea brasiliensis*	*Simpilcillium *sp.	Inactive	[63]
106	Simplicildones F	Depsidones	*Hevea brasiliensis*	*Simpilcillium *sp.	Inactive	[63]
107	Simplicildones G	Depsidones	*Hevea brasiliensis*	*Simpilcillium *sp.	Inactive	[63]
108	Simplicildones H	Depsidones	*Hevea brasiliensis*	*Simpilcillium *sp.	Inactive	[63]
109	Simplicildones I	Depsidones	*Hevea brasiliensis*	*Simpilcillium *sp.	Inactive	[63]
110	Simplicilopyrone	A-pyrone	*Hevea brasiliensis*	*Simpilcillium *sp.	Inactive	[63]
111	Botryorhodine C	Lactones	*Hevea brasiliensis*	*Simpilcillium *sp.	Weak against *S. aureus* and amethicillin-resistant *S. aureus* MIC values of 32 mg/mL	[63]
112	Licanorin	Phenolic	*Hevea brasiliensis*	*Simpilcillium *sp.	Inactive	[63]
113	3,30-dihydroxy-5,50-dimethyldiphenyl ether	Ethers	*Hevea brasiliensis*	*Simpilcillium *sp.	Against *C. neoformans* with equal MIC values of 32 mg/mL	[63]
114	Terpestacin	Terpene	*Hevea brasiliensis*	*Simpilcillium *sp.	Inactive	[63]
115	Alboatrin	Benzopyran	*Hevea brasiliensis*	*Simpilcillium *sp.	Inactive	[63]
116	(S)-dihydro-5-[(S)-hydroxyphenyl-methyl]-2(3H)-furanone	Furanones	*Hevea brasiliensis*	*Simpilcillium *sp.	Inactive	[63]
117	9-ethyl-L,7-dioxaspiro[5.5]undecan-4-ol	Fatty acid	*Hevea brasiliensis*	*Simpilcillium *sp.	Inactive	[63]
118	Cis-4-hydroxy-6-deoxyscytalone	Phenols	*Hevea brasiliensis*	*Simpilcillium *sp.	Inactive	[63]
119	4-oxo-5-phenylpentanoic acid	Fatty acid	*Hevea brasiliensis*	*Simpilcillium *sp.	Inactive	[63]
120	Methyl 5-phenyl-4-oxopentanoate	Fatty acid	*Hevea brasiliensis*	*Simpilcillium *sp.	Inactive	[63]
121	Isoevernin aldehyde	Phenolic	*Hevea brasiliensis*	*Simpilcillium *sp.	Inactive	[63]
122	Khafrefungin	C22 alkyl chain ester	Costa Rican plant sample	Unidentified *sterile* fungus	*C. albicans* with an IC50 of **0.6 nM**	[106]
123	Pestalactam D	Caprolactams	*Melaleuca quinquenervia*	*Pestalotiopsis *sp.	Inactive	[54]
124	Pestalactam E	Caprolactams	*Melaleuca quinquenervia*	*Pestalotiopsis *sp.	Inactive	[54]
125	Pestalactam F	Caprolactams	*Melaleuca quinquenervia*	*Pestalotiopsis *sp.	Inactive	[54]
126	Pestalactam A	Caprolactams	*Melaleuca quinquenervia*	*Pestalotiopsis *sp.	Inactive	[54]
127	4-o-methylpestalactam A	Caprolactams	*Melaleuca quinquenervia*	*Pestalotiopsis *sp.	Inactive	[54]
128	Tyrosol	Caprolactams	*Melaleuca quinquenervia*	*Pestalotiopsis *sp.	Inactive	[54]
129	Pestalactams B	Caprolactams	*Melaleuca quinquenervia*	*Pestalotiopsis *sp.	Inactive	[54]
130	Pestalactams C	Caprolactams	*Melaleuca quinquenervia*	*Pestalotiopsis *sp.	Inactive	[54]
131	Trichodermamide C	Amides	*Melaleuca quinquenervia*	*Pestalotiopsis *sp.	Inactive	[54]
132	3-chloro-4-hydroxyphenylacetamide	Amides	*Melaleuca quinquenervia*	*Pestalotiopsis *sp.	Inactive	[54]
133	3-chloro-4-hydroxyphenylacetic acid	phenolic acid	*Melaleuca quinquenervia*	*Pestalotiopsis *sp.	Inactive	[54]
134	(−)-Xylariamide A	Amides	*Melaleuca quinquenervia*	*Pestalotiopsis *sp.	Inactive	[54]
135	2-Hydroxy-6-Methyl-8-Methoxy-9-Oxo-9H-Xanthene-1-Carboxylic Acid	Polyketides	*Melaleuca quinquenervia*	*Pestalotiopsis *sp.	Moderate antifungal activity against *C. neoformans* and *C. gattii* (50 μM)	[54]
136	2-hydroxy-6-hydroxymethyl-8-methoxy-9-oxo-9H-xanthene-1-carboxylic acid	Polyketides	*Melaleuca quinquenervia*	*Pestalotiopsis *sp.	Inactive	[54]
137	2,8-dimethoxy-6-methyl-9-oxo-9H-xanthene-1-carboxylic acid methyl ester	Polyketides	*Melaleuca quinquenervia*	*Pestalotiopsis *sp.	Inactive	[54]
138	Pistillarin	Benzamide	*Melaleuca quinquenervia*	*Pestalotiopsis *sp.	Inactive	[54]
139	(1S,3R)-austrocortirubin	Anthraquinones	*Melaleuca quinquenervia*	*Pestalotiopsis *sp.	Inactive	[54]
140	(1S,3S)-austrocortirubin	Anthraquinones	*Melaleuca quinquenervia*	*Pestalotiopsis *sp.	Inactive	[54]
141	1-deoxyaustrocortirubin	Anthraquinones	*Melaleuca quinquenervia*	*Pestalotiopsis *sp.	Inactive	[54]
142	Austrocortinin	Anthraquinones	*Melaleuca quinquenervia*	*Pestalotiopsis *sp.	Inactive	[54]
143	Simplicildones J	Depsidones	*Hevea brasiliensis leaves*	*Simplicillium lanosoniveum*	Inactive	[64]
144	Simplicildones K	Depsidones	*Hevea brasiliensis leaves*	*Simplicillium lanosoniveum*	Against *C. neoformans* ATCC90113 with the same MIC values of 32 μg/mL	[64]
145	Globosuxanthone E	Dihydroxanthenone	*Hevea brasiliensis leaves*	*Simplicillium lanosoniveum*	Against *C. neoformans* ATCC90113 with the same MIC values of 32 μg/mL	[64]
146	(−)-Nigrosporione	Lactones	*Hevea brasiliensis leaves*	*Simplicillium lanosoniveum*	Inactive	[64]
147	(S)-dihydro-5-[(S)-hydroxyphenylmethyl]-2(3H)-furanone	Furanones	*Hevea brasiliensis leaves*	*Simplicillium lanosoniveum*	Against *C. neoformans* ATCC90113 with the same MIC values of 120 μg/mL	[64]
148	4-oxo-5-phenylpentanoic acid	Fatty acid	*Hevea brasiliensis leaves*	*Simplicillium lanosoniveum*	Against *C. neoformans* ATCC90113 with the same MIC values of 64 μg/mL	[64]
149	Isoevernin aldehyde	Phenolic acid	*Hevea brasiliensis leaves*	*Simplicillium lanosoniveum*	Inactive	[64]
150	Penicillic acid	Polyketide	*Hevea brasiliensis leaves*	*Simplicillium lanosoniveum*	Inactive	[64]
151	Botryorhodines B	Lactones	*Hevea brasiliensis leaves*	*Simplicillium lanosoniveum*	Inactive	[64]
152	Botryorhodines C	Lactones	*Hevea brasiliensis leaves*	*Simplicillium lanosoniveum*	*S. aureus* ATCC25923, methicillin-resistant *S. aureus* and *C. neoformans* ATCC90113 MIC values of 32 μg/mL	[64]
153	Simplicildones A	Lactones	*Hevea brasiliensis leaves*	*Simplicillium lanosoniveum*	*S. aureus* ATCC25923, methicillin-resistant *S. aureus* and C. neoformans ATCC90113 MIC values of 32 μg/mL	[64]
154	Simplicildones B	Lactones	*Hevea brasiliensis leaves*	*Simplicillium lanosoniveum*	Inactive	[64]
155	Coronamycin	Peptide complex antibiotic	*Monstera *sp.	Verticillate *Streptomyces* sp. MSU-2110	*C. neoformans* (ATCC 32045) **4 μg/mL**, *Pythium ultimum* 2 μg/mL, *Phytophthora cinnamomi* 16 μg/mL, *Aphanomyces cochlioides* 4 μg/mL, *Candida albicans* (ATCC 90028) 16–32 μg/mL	[34]
156	Penicilazaphilones A	Azaphilones	*Garcinia atroviridis*	*Penicillium sclerotiorum* PSU-A13	NA	[65]
157	Penicilazaphilones B	Azaphilones	*Garcinia atroviridis*	*Penicillium sclerotiorum *PSU-A13	Inactive	[65]
158	Penicilisorin	Isocoumarin	*Garcinia atroviridis*	*Penicillium sclerotiorum *PSU-A13	NA	[65]
159	Dechloroisochromophilone III	Azaphilones (oxoisochromane)	*Garcinia atroviridis*	*Penicillium sclerotiorum *PSU-A13	NA	[65]
160	Sclerotiorin	Azaphilones (oxoisochromane)	*Garcinia atroviridis*	*Penicillium sclerotiorum *PSU-A13	Moderate antifungal activity against CA and CN (MIC) values of 64 and 32 μg/mL	[65]
161	2,4-dihydroxy-6-(5,7S-dimethyl-2-oxo-trans-3-trans-5-nonadienyl)-3-methylbenzaldehyde	Phenolic acid	*Garcinia atroviridis*	*Penicillium sclerotiorum *PSU-A13	Inactive	[65]
162	( +)-(2E,4E,6S)-4,6-dimethylocta-2,4-dienoic acid	Fatty acid	*Garcinia atroviridis*	*Penicillium sclerotiorum *PSU-A13	NA	[65]
163	Flavodonfuran	Difuranylmethane derivative	*Rhizophora apiculata*	*Flavodon flavus *PSU-MA201	Inactive	[100]
164	Tremulenolide A	Sesquiterpene	*Rhizophora apiculata*	*Flavodon flavus *PSU-MA201	Against *S. aureus* ATCC25923 and *C. neoformans* ATCC90113 (MIC 128 μg/mL)	[100]
165	Hypoxylonone A	Furanones	*Cinnamomum cassia Presl*	*Hypoxylon vinosopulvinatum *DYR-1-7	Inactive	[30]
166	Hypoxylonone B	Furanones	*Cinnamomum cassia Presl*	*Hypoxylon vinosopulvinatum *DYR-1-7	Against *Lasiodiplodia pseudotheobromae* with IC50 1.01 μg/mL	[30]
167	Hypoxylonone C	Furanones	*Cinnamomum cassia Presl*	*Hypoxylon vinosopulvinatum *DYR-1-7	*L. pseudotheobromae* with IC50 value 2.40 μg/mL. medium antifungul effects on *Candida albicans*	[30]
168	(3S,8as)-3-benzyloctahydropyrrolo[1,2-A]pyrazine-1,4-dione	Pyrrolo-pyrazines	*Cinnamomum cassia Presl*	*Hypoxylon vinosopulvinatum *DYR-1-7	Medium antifungal effects on* C. albicans*	[30]
169	Cyclo(trans-4-hydroxy-l-prolyl-l-phenylalanine)	Pyrrolo-pyrazines	*Cinnamomum cassia Presl*	*Hypoxylon vinosopulvinatum DYR-1-7*	Inactive	[30]
170	Cyclo[l-(4-hydroxyprolinyl)-l-leucine]	Pyrrolo-pyrazines	*Cinnamomum cassia Presl*	*Hypoxylon vinosopulvinatum *DYR-1-7	Medium antifungal activity on *Fusarium oxysporum* with IC_50_ 10.67 μg/mL	[30]
171	(1R,4R,5R,8S)-8-hydroxy-4,8-dimethyl-2-oxabicyclo[3.3.1]nonan-3-one	MONOTERPENE lactone	*Bruguiera sexangula*	*Pestalotiopsis foedan*	Against *Botrytis cinerea* and *Phytophthora nicotianae* with MIC values of 3.1 μg/mL	[18]
172	(2R)-2-[(1R)-4-methylcyclohex-3-en-1-Yl]propanoic acid	Propanoic acid derivative	*Bruguiera sexangula*	*Pestalotiopsis foedan*	Against *Botrytis cinerea* and *Phytophthora nicotianae* with MIC 6.3 μg/mL. Modest against *C. albicans* with a MIC value of 50 μg/mL	[18]
173	Hymeglusin	Mono-/bis-alkenoic acid derivatives	*Camellia sinensis*	*Scopulariopsis candelabrum*	Against *C. albicans* showed (MIC) value of 20 μg/mL, (IC50) value (21.23 μg/mL) against *Exserohilum turcicum*	[70]
174	Fusariumesters C	Bis-alkenoic acid derivatives	*Camellia sinensis*	*Scopulariopsis candelabrum*	Inactive	[70]
175	Fusariumesters D	Bis-alkenoic acid derivatives	*Camellia sinensis*	*Scopulariopsis candelabrum*	Inactive	[70]
176	Fusariumesters E	Bis-alkenoic acid derivatives	*Camellia sinensis*	*Scopulariopsis candelabrum*	Inactive	[70]
177	Fusariumesters F	Bis-alkenoic acid derivatives	*Camellia sinensis*	*Scopulariopsis candelabrum*	Inactive	[70]
178	Acetylfusaridioic acid A	Alkenoic acid monomers	*Camellia sinensis*	*Scopulariopsis candelabrum*	Inactive	[70]
179	Fusaridioic acid D	Alkenoic acid monomers	*Camellia sinensis*	*Scopulariopsis candelabrum*	Inactive	[70]
180	Koninginins X	Polyketides	*Pedicularis integrifolia*	*Trichoderma koningiopsis* SC-5	Inactive	[39]
181	Koninginins Y	Polyketides	*Pedicularis integrifolia*	*Trichoderma koningiopsis* SC-5	Inactive	[39]
182	Koninginins Z	Polyketides	*Pedicularis integrifolia*	*Trichoderma koningiopsis* SC-5	Inactive	[39]
183	Fusaripeptide A	Cyclodepsipeptide	Roots of* Mentha longifolia*	*Fusarium *sp.	Antifungal activity toward *C. albicans*, *C. glabrata*, *C. krusei*, and *A. fumigates* with MIC of **0.11, 0.24, 0.19, and 0.14 μM**	[35, 37]
184	Adenosine	Purine nucleoside	Roots of* Mentha longifolia*	*Fusarium *sp.	Inactive	[35, 37]
185	2-((2-hydroxypropionyl)amino)benzamide	Amides	Roots of* Mentha longifolia*	*Fusarium *sp.	Inactive	[35, 37]
186	Aplojaveediins A	Polyketides	*Orychophragmus violaceus*	*Aplosporella javeedii*	*C. albicans* strain ATCC 24433, *S. aureus* sensitive (ATCC 29213) and drug-resistant (ATCC 700699)	[57]
187	Aplojaveediins B	Polyketides	*Orychophragmus violaceus*	*Aplosporella javeedii*	Inactive	[57]
188	Aplojaveediins C	Polyketides	*Orychophragmus violaceus*	*Aplosporella javeedii*	Inactive	[57]
189	Aplojaveediins D	Polyketides	*Orychophragmus violaceus*	*Aplosporella javeedii*	Inactive	[57]
190	Aplojaveediins E	Polyketides	*Orychophragmus violaceus*	*Aplosporella javeedii*	Inactive	[57]
191	Aplojaveediins F	Polyketides	*Orychophragmus violaceus*	*Aplosporella javeedii*	*S. aureus* sensitive (ATCC 29213) and drug-resistant (ATCC 700699)	[57]
192	Chetoseminudin G	Indole alkaloids	*Panax notoginseng*	*Chaetomium *sp. SYP-F7950	Inactive	[31]
193	Chetoseminudin F	Indole alkaloids	*Panax notoginseng*	*Chaetomium *sp. SYP-F7950	Cytotoxic against tumor cell line MDA-MB-231	[31]
194	Chaetocochin C	Indole alkaloids	*Panax notoginseng*	*Chaetomium *sp. SYP-F7950	Inactive	[31]
195	Chetoseminudin E	Indole alkaloids	*Panax notoginseng*	*Chaetomium *sp. SYP-F7950	Inactive	[31]
196	Dethiotetra-(methylthio)-chetomin	Indole alkaloids	*Panax notoginseng*	*Chaetomium *sp. SYP-F7950	Inactive	[31]
197	Chetomin C	Indole alkaloids	*Panax notoginseng*	*Chaetomium *sp. SYP-F7950	Against *S. aureus*, *B. subtilis*, *Enterococcus faecium* and antifungal activity against *C. albicans* (MIC) values ranging from** 0.12 to 9.6 μg mL**. cytotoxic against tumor cell lines A549 and MDA-MB-231	[31]
198	Chetomin B	Indole alkaloids	*Panax notoginseng*	*Chaetomium *sp. SYP-F7950	Inactive	[31]
199	Chetomin A	indole alkaloids	*Panax notoginseng*	*Chaetomium *sp. SYP-F7950	Cytotoxic against tumor cell lines A549 and MDA-MB-231	[31]
200	Chetoseminudin B	Indole alkaloids	*Panax notoginseng*	*Chaetomium *sp*.* SYP-F7950	Against *S. aureus*, *B. subtilis*, *Enterococcus faecium* and antifungal activity against *C. albicans* (MIC) values ranging from **0.12 to 9.6 μg mL**. Cytotoxic against tumor cell lines A549 and MDA-MB-231	[31]
201	Chetomin	Indole alkaloids	*Panax notoginseng*	*Chaetomium *sp. SYP-F7950	Inactive	[31]
202	(−)-Aureonitol	Indole alkaloids	*Panax notoginseng*	*Chaetomium *sp. SYP-F7950	Against *S. aureus*, *B. subtilis*, *Enterococcus faecium* and antifungal activity against *Candida albicans* with (MIC) values ranging from **0.12 to 9.6 μg mL**	[31]
203	Chetoseminudin C	Indole alkaloids	*Panax notoginseng*	*Chaetomium *sp. SYP-F7950	Against *S. aureus, B. subtilis, Enterococcus faecium and antifungal activity against Candida albicans* with (MIC) values ranging from **0.12 to 9.6 μg/mL**. cytotoxic against tumor cell lines A549 and MDA-MB-231	[31]
204	Ergosterol	Sterol	*Panax notoginseng*	*Chaetomium *sp. SYP-F7950	Inactive	[31]
205	Paecilin A	Dimeric chromanone	Healthy potato tissues	*Xylaria curta* E21	Against *C. albicans* ATCC 10231 with MIC of 16 μg/mL	[67]
206	Paecilins F	Dimeric chromanone	Healthy potato tissues	*Xylaria curta* E10	Against *C. albicans* ATCC 10231 with MIC of 64 μg/mL	[67]
207	Paecilins G	Dimeric chromanone	Healthy potato tissues	*Xylaria curta* E11	Against *C. albicans* ATCC 10231 with MIC of 64 μg/mL	[67]
208	Paecilins H	Dimeric chromanone	Healthy potato tissues	*Xylaria curta* E12	Inactive	[67]
209	Paecilins I	Dimeric chromanone	Healthy potato tissues	*Xylaria curta* E13	Inactive	[67]
210	Paecilins J	Dimeric chromanone	Healthy potato tissues	*Xylaria curta* E14	Inactive	[67]
211	Paecilins K	Dimeric chromanone	Healthy potato tissues	*Xylaria curta* E15	Inactive	[67]
212	Paecilins L	Dimeric chromanone	Healthy potato tissues	*Xylaria curta* E16	Against *C. albicans* ATCC 10231 with MIC of 32 μg/mL	[67]
213	Paecilins M	Dimeric chromanone	Healthy potato tissues	*Xylaria curta* E17	inactive	[67]
214	Paecilins N	Dimeric chromanone	Healthy potato tissues	*Xylaria curta* E18	Against *C. albicans* ATCC 10231 with MIC of 32 μg/mL	[67]
215	Paecilins O	Dimeric chromanone	Healthy potato tissues	*Xylaria curta* E19	inactive	[67]
216	Paecilins P	Dimeric chromanone	Healthy potato tissues	*Xylaria curta* E20	Against *C. albicans* ATCC 10231 with MIC of 64 μg/mL	[67]
217	Versixanthone F	Xanthene polyketide	Healthy potato tissues	*Xylaria curta* E22	Inactive	[67]
218	Versixanthone A	Xanthene polyketide	Healthy potato tissues	*Xylaria curta* E23	Inactive	[67]
219	Versixanthone E	Xanthene polyketide	Healthy potato tissues	*Xylaria curta* E24	Inactive	[67]
220	Massarigenin D	Lactones	*Rehmannia glutinosa*	*Massrison *sp.	Active against *C. neoformans* (16 μg/mL)	[66]
221	Spiromassaritone	Lactones	*Rehmannia glutinosa*	*Massrison *sp.	*C. albicans* (2 μg/mL), *C. neoformans* (4 μg/mL), *Tricophyton rubrum* (0.25 μg/mL) *A. fumigatus* (1 μg/mL)	[66]
222	Paecilospirone	Benzofuranes	*Rehmannia glutinosa*	*Massrison *sp.	*C. albicans* (8 μg/mL), *C. neoformans* (16 μg/mL), *Tricophyton rubrum* (2 μg/mL) *A. fumigatus* (4 μg/mL)	[66]
223	Cj-17,572	Polyketide	*Viburnum tinus*	*Pezicula *sp.	Cytotoxic, *Bacillus subtilis* DSM 10 MIC = 8.3 μg/mL, *Staphylococcus aureus* DSM 346MIC = 8.3 μg/mL, *Mucor hiemalis* DSM 2656 MIC = 4.2 μg/mL, *Pichia anomala* DSM 6766MIC = 33 μg/mL, *Schizosaccharomyces pombe* DSM 70572MIC = 33 μg/mL	[77]
224	Peziculastatin	Polyketide	*Viburnum tinus*	*Pezicula *sp.	*Bacillus subtilis* DSM 10MIC = 33 μg/mL, *S. aureus* DSM 346MIC = 16 μg/mL, *Mucor hiemalis* DSM 2656MIC = 33 μg/mL, *Rhodotorula glutinis* DSM 10134MIC = 33 μg/mL	[77]
225	Mycorrhizin A	Benzofuran	*Viburnum tinus*	*Pezicula *sp.	Cytotoxic, moderate anticandidal effect MIC = 66.6 μg/mL, active against *Bacillus subtilis* DSM 10, *Chromobacterium violaceum* DSM 30191, *Mycobacterium smegmatis* ATCC 700084, *Staphylococcus aureu*s DSM 346, *Mucor hiemalis* DSM 2656, *Pichia anomala* DSM 6766, *Rhodotorula glutinis* DSM 10134, *Schizosaccharomyces pombe* DSM 70572 MIC between 4.2 and 66 μg/mL	[77]
226	Cryptosporioptides A	Xanthone polyketides	*Viburnum tinus*	*Pezicula *sp.	Antibiofilm activity	[77]
227	Cryptosporioptides B	Xanthone polyketides	*Viburnum tinus*	*Pezicula *sp.	Antibiofilm activity	[77]
228	Cryptosporioptides C	Xanthone polyketides	*Viburnum tinus*	*Pezicula *sp.	Antibiofilm activity	[77]
229	Cr377	Pentaketide	*Selaginella pallescens*	*Fusarium *sp.	Anti-*Candidal* effect	[56]
230	Bipolamide A	Amides	*Gynura hispida*	*Bipolaris *sp. MU34	inactive	[38]
231	Bipolamide B	Amides	*Gynura hispida*	*Bipolaris *sp. MU34	Against *Cladosporium cladosporioides* FERMS-9*, Cladosporium cucumerinum* NBRC 6370*, Saccharomyces cerevisiae* ATCC 9804*, Aspergillus niger* ATCC 6275 *and Rhisopus oryzae* ATCC 1040 (MIC) values of 16, 32, 32, 64 and 64 μg/mL	[38]
232	Monoacetate Bipolamide A	Amides	*Gynura hispida*	*Bipolaris *sp. MU34	NA	[38]
233	Rubiginosin C	Azaphilones	Unidentified dead wood in Spain	*Hypoxylon rubiginosum*	Against biofilms of *C. albicans* (**> 7.8 μg/mL**) and *C. auris* (2 and 62.5 μg/mL). Non-cytotoxic	[119]
234	Rubiginosin A	Azaphilones	unidentified dead Wood in Spain	*Hypoxylon rubiginosum*	Active against biofilms of *C. albicans* and *C. auris*. Non-cytotoxic	[119]
235	Rubiginosin Z	Azaphilones	Unidentified dead wood in Spain	*Hypoxylon rubiginosum*	Active against biofilms of *C. albicans* and *C. auris*. Non-cytotoxic	[119]
236	Rubiginosin W	Azaphilones	Unidentified dead wood in Spain	*Hypoxylon rubiginosum*	Active against biofilms of *C. albicans* and *C. auris*. Non-cytotoxic	[119]
237	Rutilin A	Azaphilones	Unidentified dead wood in Spain	*Hypoxylon rubiginosum*	Active against biofilms of *C. albicans* and *C. auris*. Non-cytotoxic	[119]
238	Rutilin B	Azaphilones	Unidentified dead wood in Spain	*Hypoxylon rubiginosum*	Active against biofilms of *C. albicans* and *C. auris*. Non-cytotoxic	[119]
239	Penicolinate A	Alkaloids	Family *Poaceae* grasses in Thailand	*Penicillium *sp. BCC16054	Inactive against *Candida albicans* and *Bacillus cereus*. Active as antimalarial *Plasmodium falciparum* K-1 (3.2 μg/mL)	[28]
240	Penicolinate B	Alkaloids	Family *Poaceae* grasses in Thailand	*Penicillium *sp. BCC16054	Against *Candida albicans* 1.45 μg/mL. Active as antimalarial *Plasmodium falciparum* K-1 (1.4 μg/mL)	[28]
241	Penicolinate C	Alkaloids	Family *Poaceae* grasses in Thailand	*Penicillium *sp. BCC16054	Against *Candida albicans* 3.67 μg/mL Active as antimalarial *Plasmodium falciparum* K-1 (3 μg/mL)	[28]
242	Penicolinate D	Alkaloids	Family *Poaceae* grasses in Thailand	*Penicillium *sp. BCC16054	NA	[28]
243	Penicolinate E	alkaloids	Family *Poaceae* grasses in Thailand	*Penicillium *sp. BCC16054	NA	[28]
244	Phenopyrrozin	Alkaloids	Family *Poaceae* grasses in Thailand	*Penicillium *sp. BCC16054	Active against TB (0.0122 μg/mL), and *C. albicans* (12.4 μg/mL). Active as antimalarial *Plasmodium falciparum* K-1(3 μg/mL)	[28]
245	P-Hydroxyphenopyrrozin	Alkaloids	Family *Poaceae* grasses in Thailand	*Penicillium *sp. BCC16054	Inactive against *Candida albicans* and *Bacillus cereus*	[28]
246	Gliotoxin	Alkaloids	Family *Poaceae* grasses in Thailand	*Penicillium *sp. BCC16054	Active against TB (0.0003 μg/mL) and *C. albicans* (**1.5 μg/mL**), *B. cereus* (1.25 μg/mL). Active as antimalarial *Plasmodium falciparum* K-1 (0.4 μg/mL)	[28]
247	Bisdethiobis(methylthio)gliotoxin	Alkaloids	Family *Poaceae* grasses in Thailand	*Penicillium *sp. BCC16054	Active against TB (**0.0488 μg/mL**)	[28]
248	Occidofungin	Peptide complex Antibiotic	Soybean	*Burkholderia *sp. MS455	Against clinical *Candida* species were between 0.5 and **2.0 μg/mL**	[51]
249	Phyllomeroterpenoids A	Meroterpenes	Leaves of *A. tatarinowii* in China	*Phyllosticta *sp*.* J13-2-12Y	Inactive against *C. albicans*	[20]
250	Phyllomeroterpenoids B	Meroterpenes	Leaves of *A. tatarinowii* in China	*Phyllosticta *sp*.* J13-2-12Y	Inactive against *C. albicans*	[20]
251	Phyllomeroterpenoids C	Meroterpenes	Leaves of *A. tatarinowii* in China	*Phyllosticta *sp. J13-2-12Y	inactive against *C. albicans*	[20]
252	(S, Z)-guignardianone C	Meroterpenes	Leaves of *A. tatarinowii* in China	*Phyllosticta *sp. J13-2-12Y	Inactive against *C. albicans*	[20]
253	(S, Z)-botryosphaerin B	Meroterpenes	Leaves of *A. tatarinowii* in China	*Phyllosticta *sp. J13-2-12Y	Inactive against *C. albicans*	[20]
254	(S, Z)-phenguignardic acid methyl ester	Meroterpenes	Leaves of *A. tatarinowii* in China	*Phyllosticta *sp. J13-2-12Y	Against *S. aureus* 209P and *C. albicans* FIM709 with MIC values of **4 μg/mL**	[20]
255	(4S, 6R, 9S, 10R, 14R)-17-hydroxylated guignardone A	Meroterpenes	Leaves of *A. tatarinowii* in China	*Phyllosticta *sp*.* J13-2-12Y	Inactive against *C. albicans*	[20]
256	(4S, 6R, 9S, 10R, 14R)-guignardone B	Meroterpenes	Leaves of *A. tatarinowii* in China	*Phyllosticta *sp. J13-2-12Y	INACTIVE against *C. albicans*	[20]
257	(4S, 6R, 9S, 10S, 12S, 14R)-12-hydroxylated guignardone A	Meroterpenes	Leaves of *A. tatarinowii* in China	*Phyllosticta *sp*.* J13-2-12Y	INACTIVE against *C. albicans*	[20]
258	Palmaerones A	Dihydroisocoumarins	*Przewalskia tangutica*	*Lachnum palmae*	Moderate antibacterial against *B. cereus* and *S. aureus*. Nitric oxide (NO) production inhibitory effect 26.3 μM	[59]
259	Palmaerones B	Dihydroisocoumarins	*Przewalskia tangutica*	*Lachnum palmae*	Moderate antibacterial against *B. cereus* and *S. aureus*	[59]
260	Palmaerones C	Dihydroisocoumarins	*Przewalskia tangutica*	*Lachnum palmae*	Inactive	[59]
261	Palmaerones D	Dihydroisocoumarins	*Przewalskia tangutica*	*Lachnum palmae*	Inactive	[59]
262	Palmaerones E	Dihydroisocoumarins	*Przewalskia tangutica*	*Lachnum palmae*	Anticandidal effect 10–55 μg/mL, nitric oxide (NO) production inhibitory effect 38.7 μM and weak cytotoxicity	[59]
263	Palmaerones F	Dihydroisocoumarins	*Przewalskia tangutica*	*Lachnum palmae*	Moderate antibacterial against B. cereus	[59]
264	Palmaerones G	Dihydroisocoumarins	*Przewalskia tangutica*	*Lachnum palmae*	Moderate antibacterial against B. cereus	[59]
265	(R)-5-cholro-6-hydroxymellein	Isocoumarins	*Przewalskia tangutica*	*Lachnum palmae*	NA	[59]
266	(3R,4R)-5-cholro-4,6-dihydroxymellein	Isocoumarins	*Przewalskia tangutica*	*Lachnum palmae*	NA	[59]
267	Palmaerin A	Isocoumarins	*Przewalskia tangutica*	*Lachnum palmae*	NA	[59]
268	Palmaerin B	Isocoumarins	*Przewalskia tangutica*	*Lachnum palmae*	NA	[59]
269	Palmaerin D	Isocoumarins	*Przewalskia tangutica*	*Lachnum palmae*	NA	[59]
270	Trans-4-hydroxymellein	Isocoumarins	*Przewalskia tangutica*	*Lachnum palmae*	NA	[59]
271	Cis-4-hydroxymellein	Isocoumarins	*Przewalskia tangutica*	*Lachnum palmae*	NA	[59]
272	(R)-5-hydroxymellein	Isocoumarins	*Przewalskia tangutica*	*Lachnum palmae*	NA	[59]
273	(R)-6-hydroxymellein	Isocoumarins	*Przewalskia tangutica*	*Lachnum palmae*	NA	[59]
274	Mellein	Isocoumarins	*Przewalskia tangutica*	*Lachnum palmae*	NA	[59]
275	(R)-6-methoxy-mellein	Isocoumarins	*Przewalskia tangutica*	*Lachnum palmae*	NA	[59]
276	Persephacin	Aureobasidin derivative	Unknown plant samples	*Elsinoë *sp.	*C. auris*** 2.5 μM**, *C. tropicalis*** 0.6 μM**, *C. neoformans* **0.6 μM**, *Curvularia lunata* 0.3 μM, and *A. fumigatus* **2.5 μM**	[32]
277	Methyl 3-chloro-6-hydroxy-2-(4-hydroxy-2-methoxy-6-methylphenoxy)-4-methoxybenzoate	Polyketides	Decayed wood of *Kandelia candel*	*Nigrospora *sp. (No. 1403)	Inactive	[97]
278	(2S,5′R,E)-7-hydroxy-4,6-dimethoxy-2-(1-methoxy-3-oxo5-methylhex-1-enyl)-benzofuran-3(2H)-one	Benzofuranes	Decayed wood of *Kandelia candel*	*Nigrospora *sp. (No. 1403)	Inactive	[97]
279	Griseofulvin	Benzofuranes	Decayed wood of *Kandelia candel*	*Nigrospora *sp. (No. 1403)	Inactive	[97]
280	Dechlorogriseofulvin	Benzofuranes	Decayed wood of *Kandelia candel*	*Nigrospora *sp. (No. 1403)	Inactive	[97]
281	Bostrycin	Anthraquinone	Decayed wood of *Kandelia candel*	*Nigrospora *sp. (No. 1403)	Against *Staphylococcus aureus*, *Sarcina ventriculi*, *Bacillus subtilis*, *Pseudomonas aeruginosa*, and *Escherichia coli* with an IC50 of 3.13 μg/mL Activity against *C. albicans* with an IC50 of 12.5 μg/mL	[97]
282	Deoxybostrycin	Anthraquinone	Decayed wood of *Kandelia candel*	*Nigrospora *sp. (No. 1403)	Against *Staphylococcus aureus*, *Sarcina ventriculi*, *Bacillus subtilis*, *Pseudomonas aeruginosa*, and *Escherichia coli* with an IC50 of 3.13 μg/mL Activity against *C. albicans* with an IC50 of 12.5 μg/mL	[97]
283	5-hydroxy-3-((3′R, 5′S)-3′-hydroxy-2′-oxotetrahydrofuran-5′-yl)-7-methoxy-2-methyl-4H-chromen-4-one	Chromones	*Bruguiera gymnorrhiza*	*Trichoderma lentiforme *ML-P8-*2*	Active against *C. albicans* (50 μg/mL)	[99]
284	3-(hydroxymethyl)-5,7-dimethoxy-2-methyl-4H-chromen-4-one	Chromones	*Bruguiera gymnorrhiza*	*Trichoderma lentiforme *ML-P8-2	Inactive	[99]
285	5-hydroxy-3-(hydroxymethyl)-7-methoxy-2-methyl-4H-chromen-4-one	Polyketides	*Bruguiera gymnorrhiza*	*Trichoderma lentiforme *ML-P8-2	Active against *C. albicans* (25 μg/mL)	[99]
286	(8Z,11Z)-7-(2,4-dihydroxyphenyl)-8,12-dihydroxyhepta-8,11-dien-10-one	Phenyl derivative	*Bruguiera gymnorrhiza*	*Trichoderma lentiforme *ML-P8-2	Inactive	[99]
287	(14S,8Z,11Z)-7-(2,4-dihydroxyphenyl)-8,12,14-trihydroxynona-8,11-dien-10-one	Phenyl derivative	*Bruguiera gymnorrhiza*	*Trichoderma lentiforme *ML-P8-2	Inactive	[99]
288	Tandyukisin J	Tandyukusin derivative	*Bruguiera gymnorrhiza*	*Trichoderma lentiforme *ML-P8-2	Against *C. albicans* (25 μg/mL) and *P. italicum* ( 6.25 μg/mL)	[99]
289	Trichoharzin	Polyketides	*Bruguiera gymnorrhiza*	*Trichoderma lentiforme *ML-P8-2	Against *C. albicans* (50 μg/mL) *P. italicum* (12.5 μg/mL)	[99]
290	Tandyukisin D	Polyketides	*Bruguiera gymnorrhiza*	*Trichoderma lentiforme *ML-P8-2	Against *C. albicans* (50 μg/mL) *P. italicum* (12.5 μg/mL) *S. typhi* and *P. aerigonosa* (50 μg/mL)	[99]
291	Tandyukisin G	Polyketides	*Bruguiera gymnorrhiza*	*Trichoderma lentiforme *ML-P8-2	Against *C. albicans* (25 μg/mL) *P. italicum* (6.25 μg/mL). *S. aureas* and *P. aerigonosa* (50 μg/mL)	[99]
292	Tandyukisin C	Polyketides	*Bruguiera gymnorrhiza*	*Trichoderma lentiforme *ML-P8-2	Against *C. albicans* (25 μg/mL) *P. italicum* (50 μg/mL)	[99]
293	Aflaxanthone A	Tetrahydroxanthones	*Mangrove plant Kandelia candel*	*Aspergillus flavus *QQYZ	Against *C. gloeosporioides*, *F. oxysporum*, *F. oxysporum*, *C. musae*, and *C. albicans* with MIC values in the range of 3.13–25 μM	[105]
294	Aflaxanthone B	Tetrahydroxanthones	*Mangrove plant Kandelia candel*	*Aspergillus flavus *QQYZ	Against *C. gloeosporioides*, *F. oxysporum*, *F. oxysporum*, *C. musae*, and *C. albicans* with MIC values in the range of 3.13–25 μM	[105]
295	Fusarithioamide B	Aminobenzamide	*Anvillea garcinii (Burm.f.)*	*Fusarium chlamydosporium*	Towards *C. albicans* (MIC 1.9 µg/mL), against *G. candidum* (MIC 6.9 mg/mL), towards *E. coli* MIC 3.7 mg/mL, *B. cereus* MIC 2.5 mg/mL, *S. aureus* MIC 3.1 mg/mL	[35, 37]
296	Fusarithioamide A	Aminobenzamide	*Anvillea garcinii (Burm.f.)*	*Fusarium chlamydosporium*	Towards *C. albicans* (IZD 16.2 mm) comparable to clotrimazole (IZD 18.5 mm, positive control)	[35, 37]
297	Stigmast-4-ene-3-one	Sterols	*Anvillea garcinii (Burm.f.)*	*Fusarium chlamydosporium*	NA	[35, 37]
298	Stigmasta-4,6,8(14),22-tetraen-3-one	Sterols	*Anvillea garcinii (Burm.f.)*	*Fusarium chlamydosporium*	NA	[35, 37]
299	P-hydroxyacetophenone	Phenol	*Anvillea garcinii (Burm.f.)*	*Fusarium chlamydosporium*	NA	[35, 37]
300	Acremonisol A	Pentaketide	14 families of* Angiospermae*	*Chaetomium globosum *SNB-GTC2114	Inactive	[26]
301	Semicochliodinol A	Indole alkaloid	15 families of* Angiospermae*	*Chaetomium globosum *SNB-GTC2114	Cytotoxic towards KB (cervical uterine cancer), and MRC5 (Human lung fibroblasts)	[26]
302	Cochliodinol	Indole alkaloid	16 families of* Angiospermae*	*Chaetomium globosum *SNB-GTC2114	Against *C. albicans* (ATCC 10213) 2 μg/mL, *S. aureus* (ATCC 2921) 4 μg/mL. Cytotoxic in KB (cervical uterine cancer), and MRC5 (Human lung fibroblasts)	[26]
303	Griseofulvin	Terpene	17 families of* Angiospermae*	*Xylaria cubensis *SNB-GCI02	Cytotoxic in KB (cervical uterine cancer),	
304	Pyrrocidine C	Alkaloids	18 families of* Angiospermae*	*Lewia infectoria *SNB-GTC240	Against *S. aureus* ATCC 2921 2 μg/mL, cytotoxic in KB (cervical uterine cancer),	[26]
305	Pyrenocine A	Pyrone	19 families of* Angiospermae*	*Lewia infectoria *SNB-GTC240	Inactive	[26]
306	Novae zelandin A	Pyrone	20 families of* Angiospermae*	*Lewia infectoria *SNB-GTC240	INACTIVE	[26]
307	Alterperylenol	Diterpene	21 families of* Angiospermae*	*Lewia infectoria *SNB-GTC240	Against *S. aureus* (ATCC 2921) 32 μg/mL	[26]
308	Drimenol	Sesquiterpene	*Macrotermes natalensis *colonies	*Termitomyces*	*C. albicans* (32 μg/mL), *C. auris* (30 μg/mL), *A. fumigatus* (8 μg/mL), *C. krusei* (32 μg/mL), *C. neoformans* (8 μg/mL), *C. glabrata* (30 μg/mL)	Kruzenbeck et al. 2023

Bold values represent the most potent activities against top priority pathogenic species

##### 2. Alkaloids

**Fig. 2:**
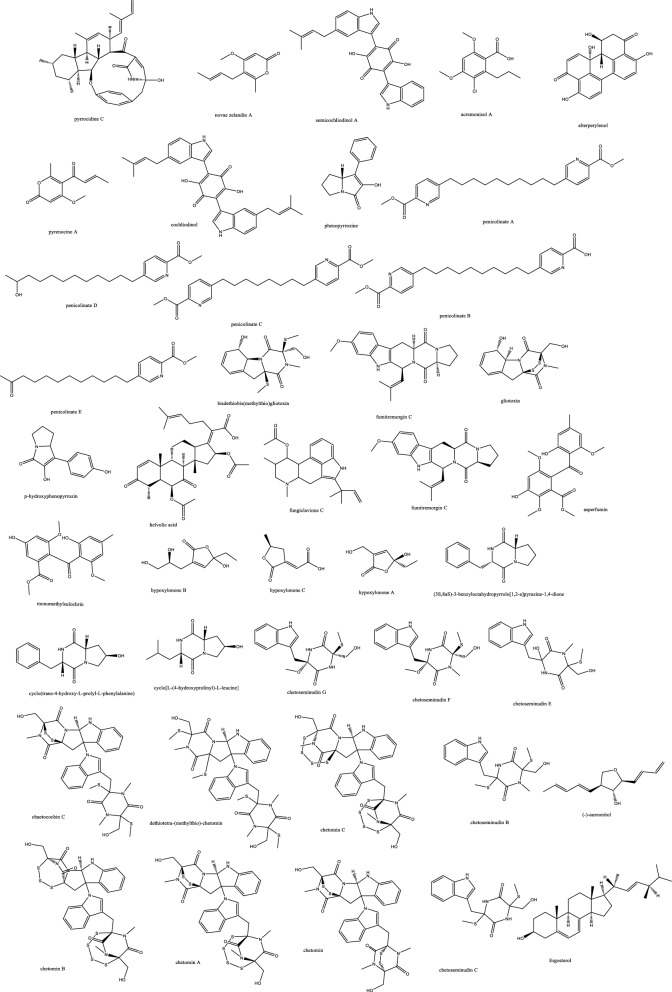
Endophytic alkaloids with antifungal potential activity

Cochliodinol was isolated from the endophyte *Chaetomium globosum* SNB-GTC2114 and demonstrated anticandidal effect of 2 μg/mL as well as potent cytotoxicity with IC_50_ as low as 0.53 μM in cell lines KB, MRC5, and MDA-MB-435 [26]. Cochliodinol is a prenylated dimeric indole alkaloid first described in 1975 by Brewer et al. and isolated later from several *Chaetomium* species [27]. The pyridine derivatives penicolinates A–C isolated from *Penicillium* sp. BCC16054 showed moderate activity against *C. albicans* compared to amphotericin B whose IC_50_ value of 0.072 μg/mL. Despite their antimalarial and antitubercular effects, they manifested cytotoxicity against NCI-H187, MCF-7, KB, and the normal VERO cell lines, which might retard the progress of these molecules to the clinical use [28]. The benzophenone asperfumoid and indole bioactive mycotoxin alkaloids were isolated from *Cynodon dactylon* endophytes and revealed marked anti-candidal activity. Helvolic acid and physcion fermentations were optimized to provide large scale cultures with an activity in the range of MIC 31-125 μg/mL [29] (Fig. 2). *Hypoxylon* species from *Cinnamomum cassia* Presl biosynthesized three furanones and three pyrrolo-pyrazines with hypoxylonone C and (3S,8aS)-3-benzyloctahydropyrrolo[1,2-α]pyrazine-1,4-dione exerting a marked anticandidal effect [30]. Indole alkaloids with notable activity against *C. albicans* were isolated from *Chaetomium* sp. SYP-F7950 endophyte with MIC range of 0.12 to 9.6 μg/mL although not devoid of cytotoxicity in A549 and MDA-MB-231 cell lines [31].

##### 3. Peptides

**Fig. 3:**
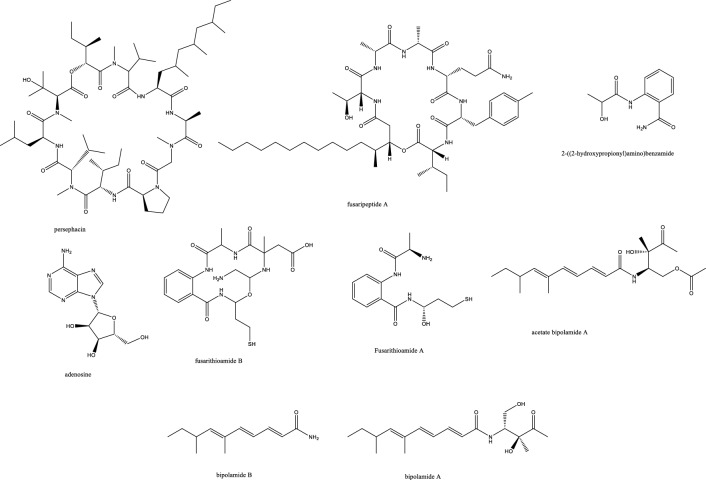
Endophytic peptides and amides with antifungal potential activity

Among the few effective antifungal molecules towards *C. auris* is persephacin, which was isolated from some plant-endophytes. This cyclic peptide was described as an aureobasidin like structure devoid of phenylalanine but possessing persephanine as an unusual amino acid. Persephacin exerted a significant activity against fluconazole-resistant *C. albicans* and *A. fumigatus causing eye infection in an ex-vivo study, which outperformed control drugs* [32] (Fig. 3). Moreover, the 3D tissue models, highly simulating in-vivo studies, demonstrated its safety for treatment of eye infection with negligible irritation or toxicity [32].

Around four-hundred endophytes of *Eugenia bimarginata* DC were isolated and examined for their antifungal efficacy against *Crypotcoccus neoformans and gattii,* which resulted in discovering *Mycosphaerella sp.* UFMGCB 2032 extract with MIC values of 31.2 μg/mL and 7.8 μg/mL [33]. Upon inspecting its two major compounds, the eicosenoic acid derivative possessed an extra double bond, which was believed to alter the Log P value and alter receptor interaction. Myriocin reduced fungal virulence by stimulating the production of *Cryptococcal* pseudo hyphae, and both compounds showed synergistic effect with amphotericin B and might induce apoptotic cell death in fungi [11]. Coronamycin, the complex mixture of bioactive peptides was effective with a lower MIC value than flucytosine against *C. neoformans*, yet it exhibited negligible activity towards several fungal strains as *A. fumigatus, A.* o*chraceus, Fusarium solani, Rhizoctonia solani and Candida* species as *C. parapsilosis* (ATCC 90018), *C. krusei* (ATCC 6258), *C. tropicalis* (ATCC 750) except *C. albicans* (ATCC 90028) [34]. A cyclodepsipeptide comprised of six amino acids and a long chain fatty acid was isolated from *Fusarium* sp. inhabiting the roots of *Mentha longifolia* and displayed potent antifungal effects against three *Candida* species as well *A. fumigatus*. The antimalarial activity was pronounced against *P. falciparum* (D6 clone) with MIC value of 0.34 μM; however, its cytotoxicity in cell lines L5178Y and PC12 might hinder further progression [35].

##### 4. Amides

The amino benzamide derivatives, fusarithioamide B and A [36]. manifested potent activity *C. albicans* compared to the standard antifungal clotrimazole, but their selective cytotoxicity against KB, HCT-116, BT-549, SKOV-3, SK-MEL, and MCF-7 cell lines might require chemical optimization to be suitable for further in-vivo and clinical studies. The proposed mode of action is possibly due to their sulphur-based structure reported before to react with SH-moieties in bacterial and microbial proteins and disrupting their metabolism [37]. Of the three isolated decatriene fatty acid amides, only bipolamide B was moderately active with broad spectrum against several fungal cells. The structural resemblance allowed prospecting a role of the five membered carbon short chain in bipolamide A to control toxicity/activity ratio since it was completely ineffective [38].

##### 5. Polyketides

The second was koninginins X–Z polyketides from *Trichoderma koningiopsis* SC-5 with no demonstrated activity up to 100 μg/mL against *C. albicans* [39]. The endophytic fungus *Aspergillus* sp. AP5 isolated from *Phragmites australis* was chemically profiled to unveil the antifungal activity of its ethyl acetate crude extract towards *C. albicans* ATCC 10231 *and A. niger.* Nafuredin, carbonarin A and I, and yanuthone D were detected by HR-LCMS and prospected to be the bioactive antifungal ingredients according to PASS software of molecular networking [40]. Pestafolide A, the reduced azaphilone derivative isolated from the endophyte *Pestalotiopsis foedan* in China showed activity against *Aspergillus fumigatus* (ATCC 10894). This azaphilone structure partially resembled decipinin A [41] in the two spiro connected pyran rings and resembled monascusone A [42] in its partial tetrahydroisochromenone moiety, yet monascusone A lacked the C-9 tetrahydropyran. Other isobenzofuranones were isolated as pestaphthalides A and B, closely related to acetophthalidin [43], with antifungal effect against *Candida albicans* (ATCC 10231) and *Geotrichum candidum* (AS2.498), respectively (Table 1) [44]. Pestaphthalides A and B were totally synthesized before through iridium-aryl borylation followed by a Suzuki-cross coupling/Jacobsen-epoxidation, epoxide opening and a rearrangement of cyclic carbonate/γ-lactone [45]. Biosynthetically, azaphilones originate from a NR-PKS polyketide and fatty acid pathway combination occasionally involving amino acids [46]. Pestalofones are derived from a terpenoid/polyketide pathway with structural similarity to iso-A8277C isolated before from the endophyte *Pestalotiopsis fici* [47, 48]. Pestalofones B and C originated from the Diels–Alder reaction of two molecules of iso-A82775C with a characteristic polyhydroxylated cyclohexane ring either spiro connected or via exocyclic methylene. *A. fumigatus* (ATCC 10894) was susceptible to pestalofones C and E with MIC values of 1.10 and 0.90 μM, respectively [49]. The NRPS/PKS biosynthesized occidiofungin obtained from the soyabean endophyte *Burkholderia* sp. MS455 inhibited the growth of *A. flavus* by stimulating apoptotic cell death [50]. Occidofungin demonstrated a potent antifungal activity against several *Candida* clinical isolates including those with fluconazole and caspofungin resistance. According to the time-kill and PAFE assays, the target of occidiofungin was presumably different from caspofungin and echinocandin. Furthermore, it showed gastric acid and temperature stabilities, which predispose its possible suitability for oral route administration than caspofungin after conducting bioavailability studies. With only azoles till now as the approved oral antifungal agents, in-depth studies of occidofungin are highly warranted [51]. More polyketides phomopoxides of the cyclohexenoid polyhydroxylated type were isolated from the *Phomopsis* sp. YE325 endophyte with unique stereochemical and oxygenation patterns. Similar hexenoids were reported from *Streptomyces, Eupenicillium and Aspergillus* before [52]*.* Phomopoxides B, D and G revealed a significant antifungal activity against *C. albicans and A. niger* [53]. Among a large-scale library, isolated from *Pestalotiopsis* sp., comprised of caprolactams, polyketides, quinones, and polamides only 2-hydroxy-6-methyl-8-methoxy-9-oxo-9H-xanthene-1-carboxylic acid reported a weak anticryptococcal activity of 50 μM [54]. Comparable to nystatin, CR377 represented a potent selective anticandidal molecule [55]. CR377 was first isolated from unidentified *Fusraium* sp. and later obtained from *Fusarium fujikuroi* by Von Bargen et al. who identified the genetic cluster and renamed it as fujikurin A [56]. Aplojaveediins A isolated from the endophyte *Aplosporella javeedii* exhibited antifungal effect 100 μM when tested against *C. albicans ATCC 24433* hyphal forms and *Saccharomyces cerevisiae* while being non-cytotoxic towards cancer cell lines HUH7, THP-1, and CLS-54. Additionally, it showed a fungicidal activity and a fast viability decline when given in a fourfold MIC value compared to hygromycin, which only exerted a static growth inhibitory effect [57].

**Fig. 4:**
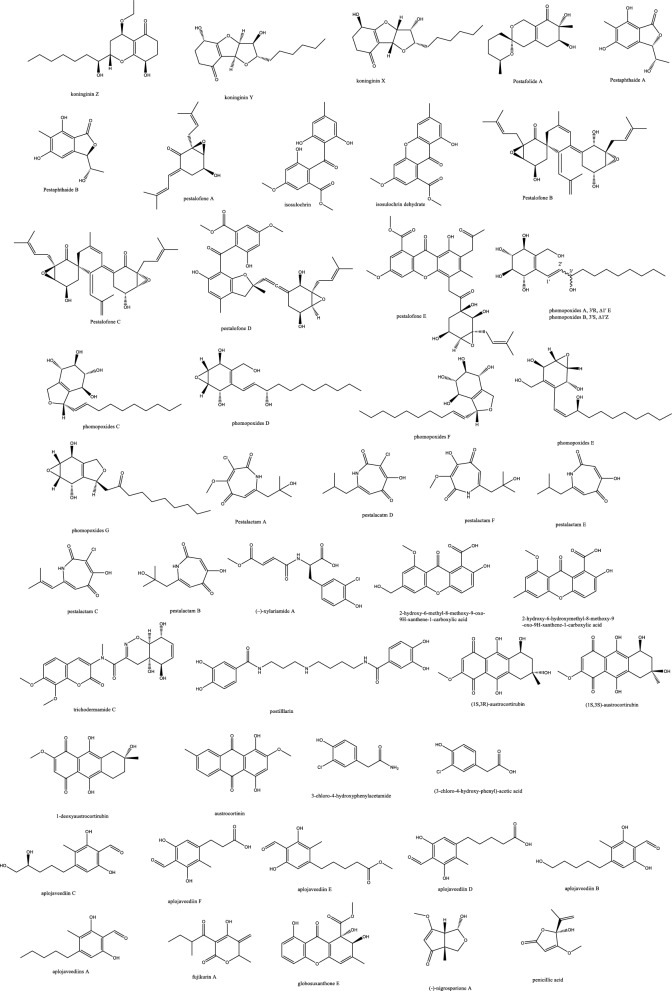

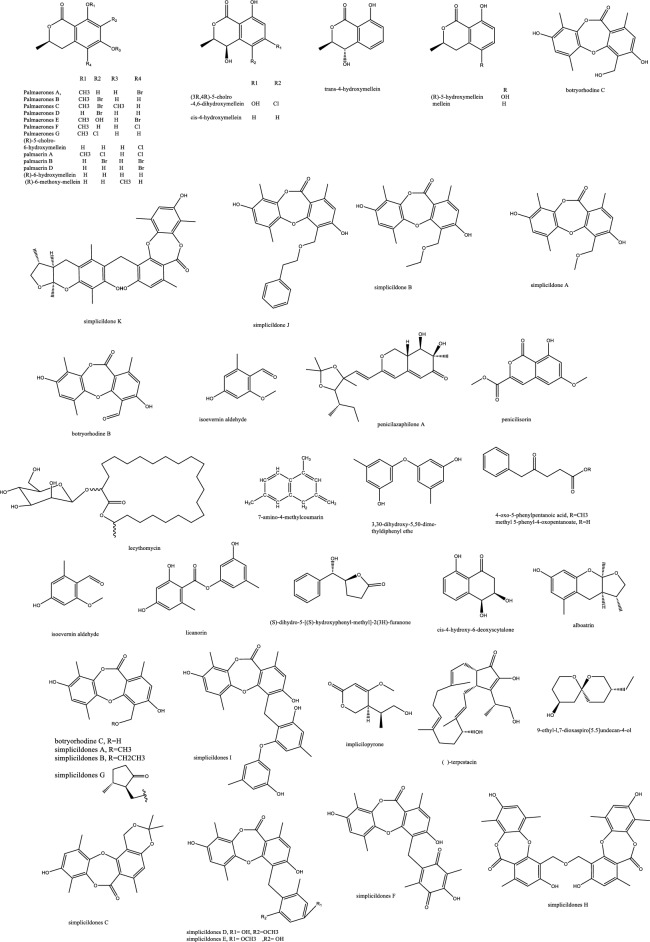

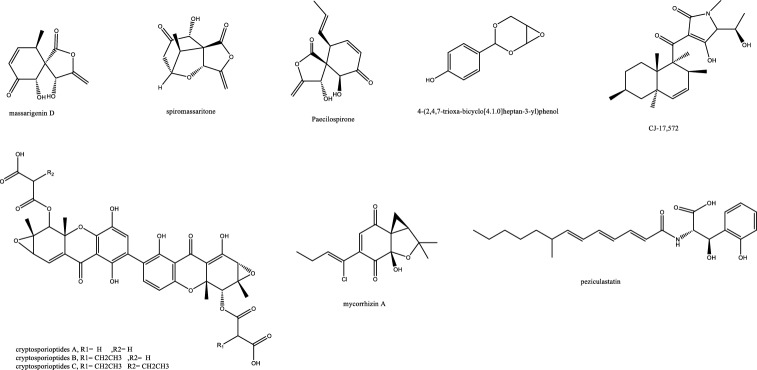
Endophytic polyketides with antifungal potential activity

Halogenated fungal derived compounds were not subjected to sufficient scrutinization as antifungal agents, and few reports stated their dominant sources from marines, sponges and algae. Moreover, questions remained unanswered about their enzymatic or non-enzymatic biosynthesis to better manipulate this potential source of underexplored compounds [58]. In a recent study, histone deacetylase (HDAC) inhibitors as suberoylanilide hydroxamic acid were employed to enhance the isocoumarin biosynthetic pathways in *Lachnum palmae* and resulted in the production of brominated and chlorinated products with moderate activity against *B. cereus* and *S. aureus* although with insignificant effect against *C. albicans and C. neoformans. *Zhao et al. noted the higher activity of the brominated molecules compared to the chlorinated one [59]. In accordance with the host plant activity, the fungal endophyte *Botryosphaeria rhodian* yielded the depsidones Botryorhodines A and B. Both the compounds and the crude extract manifested potency against *A. terreus* human pathogen, possibly attributed to the aldehydic group of C-3 position. Depsidones from natural products were reported typically from lichens and few were found in plants or endophytes [60]. The previously synthesized 7-amino-4-methylcoumarin was obtained in decent amounts from the endophytic *Xylaria* sp. YX-28 residing in an ancient 1000-year-old Ginkgo tree. The abundance and large-scale production of this wide spectrum antimicrobial and antifungal agent warranted more exploitation; especially for priority pathogens as *C. albicans* and *A. niger* [48]. The rare in nature macrolactone glycoside Lecythomycin exerted a moderate inhibitory but selective effect towards the growth of *A. fumigatus and C. kruzei* since it manifested no similar action on closely relevant strains as *C. albicans and A. faecalis or bacteria.* This was credited to its uncommon 24-membered lactone and the mannoside sugar part, only ascribed to few fungi before [61]. The isocoumarin cladosporin obtained in high titer amount of 24% from *Cladosporium cladosporioides* was shown to be active against *Plasmodium falciparum* in the nanomolar range and against *Cryptococcus neoformans.* The chemical features of cladosporin were analyzed to highlight the importance of the open unsubstituted 5′-position, C-6’ R configuration, and C-6 hydroxylation for the antifungal activity [62]. Depsidones as simplicildone C was isolated from *Simplicillium* sp. PSU-H4 in Thailand and displayed a weak antifungal effect against *C. neoformans* with a high safety profile towards VERO cell lines, which suggested the need to improve this depsidone nucleus and enhance its potency by medicinal chemists [60, 63]. In the same way, simplicildones K and globosuxanthone E produced by *Simplicillium lanosoniveum* were active against *Cryptococcus neoformans* ATCC90113 with the MIC value of 32 μg/mL [64]. Both the polar and nonpolar fractions of the endophytic fungus extract *P. sclerotiorum* PSU-A13 manifested good antimicrobial and anti-HIV integrase activities. Contrarily to what might be considered, the assays conducted on three azaphilone acetonide, deacetonide and isocoumarin nuclei showed the significance of the chlorine atom in the sclerotiorin isolated from the hexane extract for both the antifungal and anti-HIV effects, irrespective of the azaphilone unit [65] (Fig. 4). The novel skeleton of spiro 5, 6 membered lactones revealed remarkable antifungal effect against both *C. albicans and C. neoformans* with MIC_80_ values down to 2 and 4 μg/mL, respectively*.* For instance, spiromassaritone isolated from *Rehmannia glutinosa* endophytes was more potent than griseofulvin by 3 folds magnitude [66]. Dimeric chromanones showed potent effect against *C. albicans* ATCC 10231 with paecilins A the most active among its congeners. Similarly, strain *Escherichia coli* ATCC 25922 was inhibited by paecilins L and N with MIC values of 16 μg/mL for each, and *Salmonella enteritidis* ATCC 25923 were susceptible to chromanones paecilins L and N with MIC values 32 μg/mL for each [67]. The crude endophytic extract of *A. tubingensis* AN103 demonstrated higher antifungal effect than its pure compounds with MIC values between 3.2 and 14 μg/mL against *F. solani* MLBM227, *A. niger* ATCC 16404*, C. albicans* ATCC 10231, and *A. alternata* MLBM09. These compounds were the naptha-γ-pyrones pyranonigrin A, TMC 256 A1 as well as fonsecin and asperazine [68].

##### 6. Fatty acid derivatives

*Candida albicans* infections were characterized as serious health threats with more than three hundred thousand infected cases reported per year. Women suffer from vulvovaginal candidiasis, which is a recurrent infection and at least once in life 75% of females encountered it. Immunocompromised patients are particularly vulnerable where mortality rate can reach up to 50% even with drug treatment [69]. In two attempts to study endophytes from food sources in China, tee tree endophyte *Scopulariopsis candelabrum* was fermented in large scale to obtain monomers and dimers of alkenoic acids namely, hymeglusin and fusariumesters, and the former showed anticandidal activity with MIC 20 μg/mL [70]. From rare liverworts as *Scapania verrucosa* Heeg, which are difficult to obtain in large amounts, endophytes represent a promising way to study secondary metabolites due to their high biomass production. For example, *Chaetomium fusiforme* was isolated from *S. verrucosa* and produced several volatile molecules, mainly methyl ester (21.25%), acetic acid (35.05%), 3-methyl-, and butane-2, 3-diol (12.24%), and valeric acid, possibly causing its effect against *Candida albicans* ATCC76615*, Cryptococcus neoformans* ATCC32609*, Trichophyton rubrum,* and *Aspergillus fumigatus* with IC_80_ values of 32, 64,64 and 8 μg/mL, respectively [71].

##### 7. Miscellaneous

**Fig. 5:**
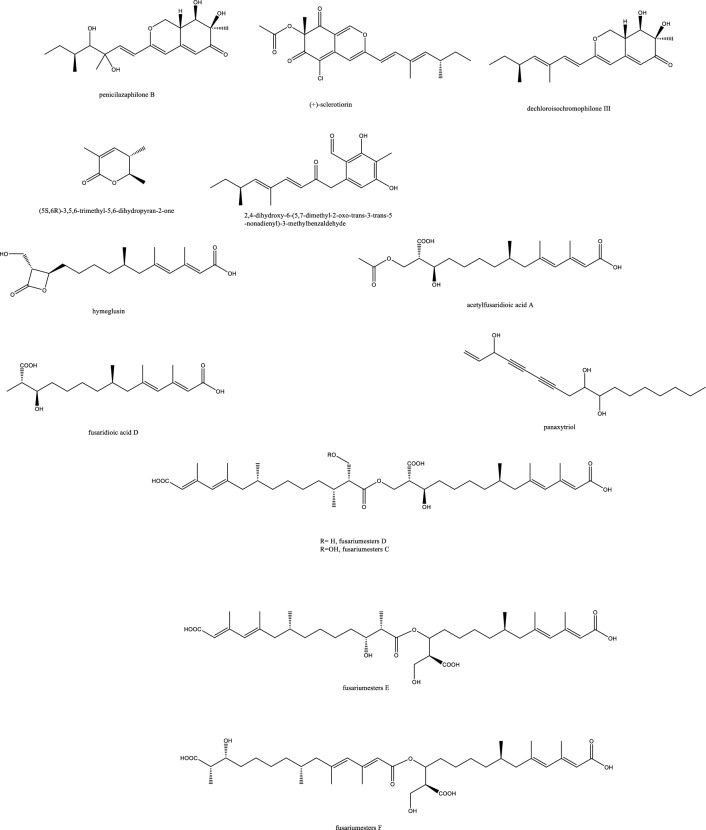
Endophytic miscellaneous compounds with antifungal potential activity

**Fig. 6:**
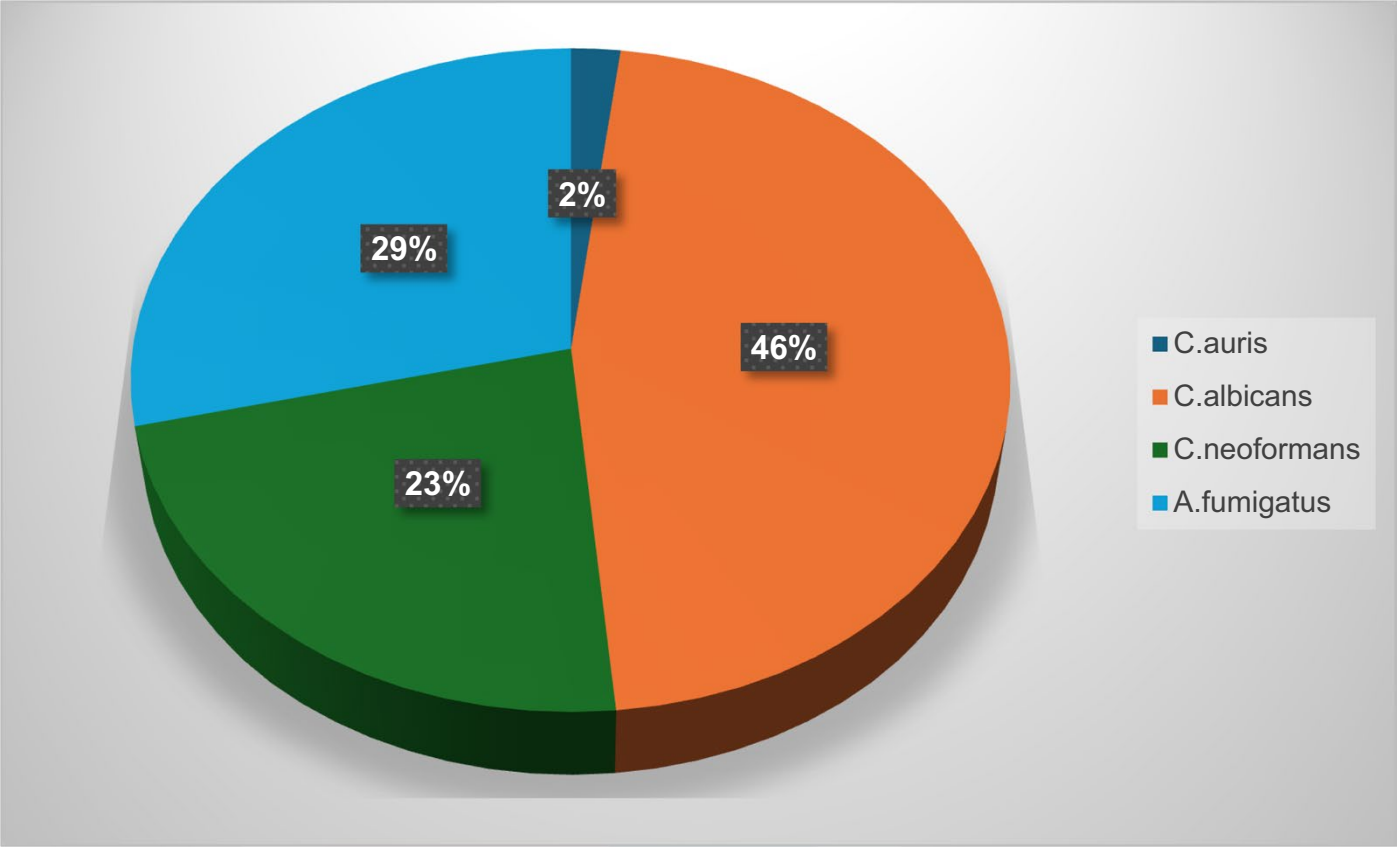
Percentage of endophytic isolated compounds with promising activity against selected priority pathogens (total 101 compounds)

Several ascomycetes endophytic fungi were isolated from family Cupressaceae hosts as *Cupressus, Platycladus, and Juniperus* species in Iran and revealed anti-aspergillosis activity against human pathogenic *Aspergillus fumigatus* IFRC460 and *Aspergillus niger* IFRC278 through Petri dish dual-culture assays. The aryl ethers aspergillethers A and B were isolated from a *Pulicaria crispa* Forssk endophyte and reported significant activity against *C. albicans and Geotrichium candidum* [72, 73]. A diphenyl ether namely, 4-dihydroxy-2′, 6-diacetoxy-3′-methoxy-5′-methyl-diphenyl ether was isolated from *Verticillium* sp. and showed significant antifungal effect against *C. albicans and A. fumigatus* but not *Cryptococcus neoformans* [74]. Mycorrhizin A was first isolated from a mycorrhizal fungus of *Monotropa hypopitys* L. [75], and several attempts of synthesis were conducted before its complete synthesis in 1982 [76]. This benzofuran reported broad spectrum antimicrobial effect with a moderate activity towards *C. albicans* [77]. *Mycosphaerella* sp was isolated from *Eugenia bimarginata* and provided two usnic acid derivatives, mycousfuranine and mycousnicdiol, displaying moderate activity against *Crypotcoccus neoformans and gattii 50 and 250* μg/mL [78] (Figs. 5 and 6).

##### 8. Endophytic bioactive extracts

**Fig. 7:**
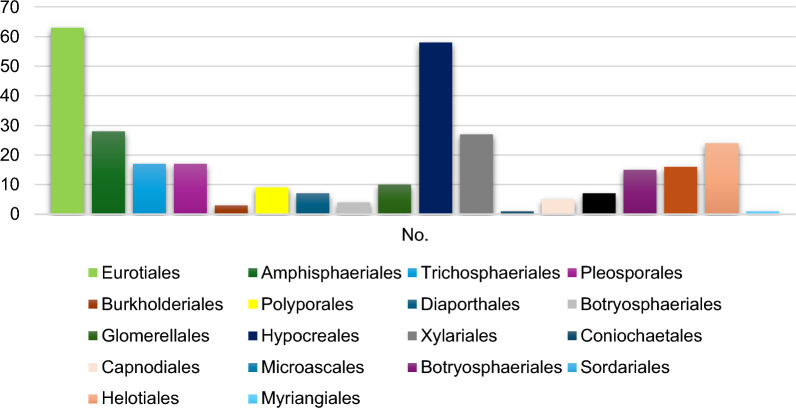
Endophytic fungal diversity with potential antifungal effect against selected priority pathogens. (Y-axis: number of endophytic fungal strains with antifungal activity against selected strains, X-axis: diversity of the endophytic fungal strains)

The endophyte *Epicoccum nigrum* isolated from the roots of *Maxillaria rigida* was in a study among more than 383 isolated endophytes in Brazil and displayed activity against both *C. albicans and C. krusei* with MIC values of 7.8 μg/mL. No further work was done to investigate the extract and isolate the bioactive components [79]. The fungal isolates obtained from several arid plant species cultivated in Andalusia were examined for their secondary metabolite production, which was enhanced using polymeric resins such as Amberlite® and Diaion®. For instance, calbistrin A, dextrusin B4, and secalonic acid C were produced exclusively in presence of XAD-16 resin from *Psudocamarosporium* sp., *Alternaria* sp. and *Sclerostagonospora* sp. CF-281856, respectively. About 61 fungal extracts reported 70% inhibition of *A. fumigatus* ATCC 46645, and 23 fungal strains were effective against *Candida albicans* MY1055 [80] (Fig. 7). The amazonian plant *Arrabidaea chica* was the source of more than 100 endophytes whose ethyl acetate extracts were active against different microbial strains. The most active was *Botryosphaeria mamane* CF2-13 extract against *P. mirabilis, E. coli, S. enterica, S. epidermidis, B. subtilis, S. marcescens,, A. brasiliensis,, C. tropicalis, K. pneumoniae, C. albicans, S. aureus and C. parapsilosis* with MIC values in the range of 0.3 mg/mL [81]. The endophytes from *Monarda citriodora* Cerv. ex. Lag extended the antifungal effect of its host plant and showed biocontrol ability and complete inhibition against strains *Sclerotinia sp., Aspergillus flavus, A. fumigatus* and *Colletotrichum capsica* using dual culture assays with 50% inhibition ranging between 54 and 100% [82]. Four endophytic strains isolated from *Dendrobium devonianum* and *D. thyrsiflorum* cultivated in Vietman demonstrated weak antifungal effect towards *A. fumigatus* and *C. albicans* using agar diffusion assay [83]. More than nine ginkgo endophyte extracts proved potency against *C. albicans, and A. fumigatus Trichophyton rubrum, and Cryptococcus neoformans* and as antioxidants when tested by DPPH assay [84]. From the orchids trees *Dendrobium devonianum and D. thyrsiflorum,* more than 25 endophytic isolates were purified and identified based on ITS sequencing, and *Fusarium, Epicoccum, and Phoma* species were the dominant strains from both roots and stems, yet none exerted notable effect upon *C. neoformans* despite pronounced antibacterial effect against *Bacillus subtilis, Escherichia coli,* and *Staphylococcus aureus* [83]. 44.8% of endophytes of *Pseudolarix kaempferi* were screened against *Pyricularia oryzae* P-2b model and exhibited activity towards Cryptococcus neoformans, *Trichophyton rubrum, and Candida albicans* demonstrated by either conidia inhibition, swelling of hyphae or beads formation [85]. Moreover, the VOCs of family Cupressaceae endophytes showed time dependent inhibition of *A. niger and A. fumigatus* fungal growth in less than a week. The most active of which were *Trichoderma koningii* CSE32 and *T. atroviride JCE33 extracts* [7].

##### 9. Mode of action of antifungal natural products

Unlike antibacterial compounds, many of the tested antifungal molecules are still far from being deeply studied to illustrate their exact mode of action, yet some mechanistic views were presented regarding terpenoids as follows. Fungal cell wall components include polymers such as chitin, mannoproteins, sphingolipids and glucans. While glucans are dominated by 1,3 or 1,6-glucose polymers in the cell wall, ergosterol constitutes the cell membrane. Inhibition of these polymers results in cellular death [86]. Many natural products act by crossing fungal cell walls and accumulating inside the lipid bilayer. This is true for terpenes/terpenoids whose lipophilicity enhance their capacity to go into the cell membrane and either lead to cell death or lack of germination [87]. Terpenes were reported to act by suppressing energy generation through mitochondrial damage. This alters virulence functions, cell wall protection and ergosterol biosynthesis, which is essential for fungal integrity [88]. Change in membrane hyperpolarization affects permeability and ions as calcium, pumps, and ATP pool eventually leading to cellular apoptosis. These events can be assessed by evaluating mitochondrial membrane permeability (MMP) and the amount of H + pumped out of the mitochondrial matrix [89]. A major fungal strategy to develop resistance is through efflux pumps, which remove substances out of the cell and undermine the effect of accumulated antifungal agents [90]. Many terpenes can act by inhibiting efflux pumps to cut down fungal resistance by down-regulating genes coding for efflux pumps as CDR1 and CDR2. Drimenol and other drimane sesquiterpenes were isolated from genus *Termitomyces were screened against C. albicans and fluconazole resistant strains of C. parapsilosis, C. glabrata, C. albicans, C. krusei, Aspergillus, Cryptococcus and C. auris* revealed potent microbicidal effect with MIC value of drimenol was investigated further and showed a fungal cell, membrane damaging effect. Further studies with mutant spot assay manifested changes in pathways and genes as the Crk1 kinase associated gene products, orf19.759, orf19.1672, Ret2, Cdc37, and orf19.4382 [91]. This was further assisted with heterozygous barcoded mutant collection assay, which was conducted on both Saccharomyces cervesise and *C. albicans* to unveil the target genes and complemented with molecular docking [92].

#### III. Actinomycete endophytes with activity against selected pathogens

*Streptomyces* YHLB-L-2 was isolated among 269 endophytes from medicinal plants in Fenghuang Mountain and its yeast peptone media fermentation produced quinomycin A, which was active against *Cryptococcus neoformans* and clinical resistant strains of *Aspergillus fumigatus* [93]. Endophytic *Streptomyces* in *Arnica montana* L. produced the cycloheximide with anticandidal effect against *C. parapsilosis, presumably* produced for the benefit of the host plant as antifungal and antiviral [94]. Streptomyces endophyte from roots of wheat plant hindered the growth of *Aspergillus niger* MTCC 282, despite showing no chitinase production, which indicated that its secondary metabolites could elicit the antifungal effect [95]. As many other *Streptomyces* strains, Streptomyces sp. K-R1 associated with root of *Abutilon indicum* produced the anthranilic peptide actinomycin D, yet it revealed crude extract weak activity towards fluconazole resistant *Candida albicans MTCC-183* and *Aspergillus niger MTCC-872* with MIC 1 mg/mL [96].

### B. Marine-derived endophytes with activity against selected pathogens

**Fig. 8:**
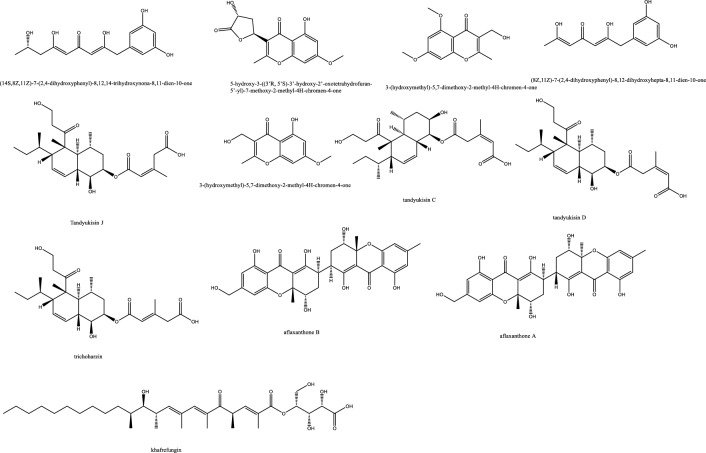
Marine endophytic compounds with potential antifungal activity against selected priority pathogens

Bostrycin and its deoxy derivative were isolated from the marine endophyte *Nigrospora* 1403 and reported moderate activity towards *C. albicans*, yet they were both of potent cytotoxic potential. The MTT assay of bostrycin suppressed the growth of six cancer cell lines, MCF-7, Hep-2, A549 Hep G2, KB, and MCF-7/Adr with IC_50_ values of, 6.13, 5.39, 2.64, 5.90, 4.19, and 6.68 μg/mL, respectively. Similarly, deoxybostrycin inhibited the growth of all tumor cells with IC_50_ between 2.4 and 5.4 μg/mL [97]. The marine macroalgae in bay of fundy in Canada provided about seventy-nine different endophytic species isolated from ten hosts. Among them were *Penicillium *sp,* Helicomyces *sp.,* Aspergillus *sp.,* Botrytis *sp.,* Trametes versicolor, Coniothyrium *sp.,* Cladosporium *sp.,* Coelomycete I, Hypoxylon *sp.,* and Botryotinia fuckeliana. The* mycelial and media methanol extracts displayed significant activity against *C. albicans, P. aeruginosa and S. aureus* [98]. Polyketides of the tandykusin type as well as phenyl derivatives were isolated from the mangrove endophyte *Trichoderma lentiforme* ML-P8-2 and exerted a moderate effect against *C. albicans* [99]. The sesquiterpene tremulenolide A was isolated from the endophyte *Flavodon flavus* PSU-MA201 together with a rare yet inactive difuranyl methane. Tremulenolide A was mildly active against *C. neoformans* [100] (Fig. 8). Heterodimeric xanthones with a 7,7′-Linkage were isolated from the mangrove plant endophyte *Aspergillus flavus* QQYZ. The non-biaryl linkage was reported for the first time in xanthones and possibly played a pronounced role in the broad-spectrum antifungal effect of aflaxanthone A and B. Other endophytic xanthones with C3-N-C2′ bridge like incarxanthone F [101], 2,2′-biaryl bond like phomoxanthones C–E [102], 2-4′-linkage like penicillixanthone B [103] or 4,4′-linkage like deacetylphomoxanthone C [104] were recorded before from *Peniophora incarnata, Phomopsis sp*. xy21, *Setophoma terrestris* (MSX45109), *Phomopsis *sp. HNY29-2B, respectively, but showed no activity against fungi [105]. From an unidentified endophytic fungus in Costa Rica, khafrefungin was separated and found effective against both filamentous fungi and yeasts; particularly, *C. albicans, C. neoformans* with MFC of 4 and 4 mg/mL respectively. In this scenario, complex sphingolipids were lost with the inhibition of phosphoinositol transfer to ceramides [106]. Khafrefungin specifically hindered inositol phospho-ceramide (IPC) synthase without inhibiting mammalian sphingolipid synthesis, which added to its safety profile.

### C. Coculture techniques of endophytes with activity against selected pathogens

Under standard fermentation procedures, microbial chemical diversity is usually limited, and the rediscovery of known metabolites becomes a common scenario, which delays novel molecules drug discovery [107]. Coupled with the large number of uncultivable strains, already existing in environment but not accessible to research, the use of different fermentation strategies to unlock the power of cryptic metabolites seems mandatory. For example, one strain many compounds (OSMAC)technique where a single promising microbial strain is subjected to different media and culturing conditions to maximize its production of bioactive molecules. Another example is epigenetic methods and employing modifiers like DNA methyl transferase inhibitors or histone deacetylase inhibitors to manipulate genetic clusters and possibly activate the transcription of secondary metabolite silent genes. Lastly, coculturing where the metabolites of one strain can induce the expression of another strain metabolites [108]. It is worth mentioning that coculturing strategies are usually straightforward and effective with no need for genetic level operations. In this section, data will be presented about how cocultures inspired the discovery of antifungal molecules against WHO priority pathogens [109].

**Fig. 9:**
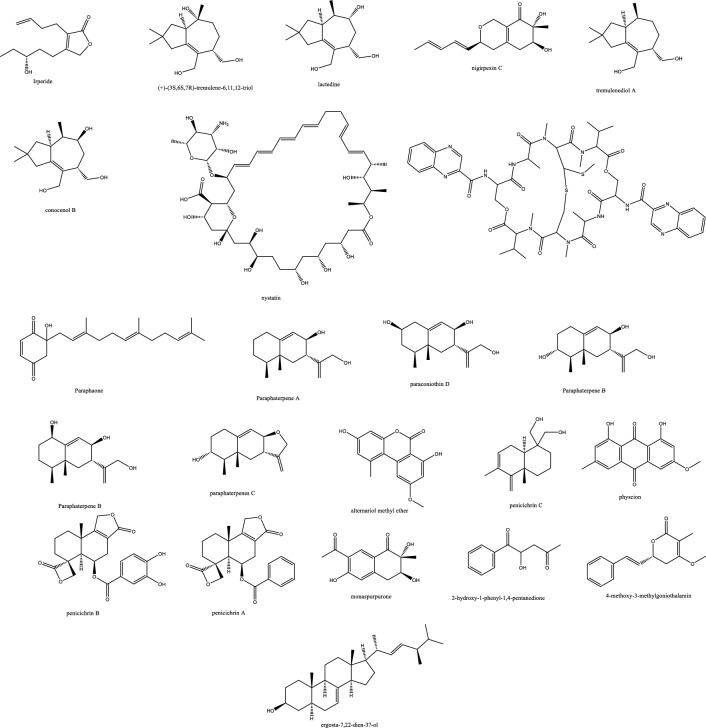
Coculture endophytic compounds with potential antifungal activity against selected priority pathogens

The coculture of *Cophinforma mamane with C.albicans* shed light on the nature of interaction between the two microbes, particularly, by applying untargeted metabolomics and UPLC-MS–MS analysis to identify the compounds produced in both the axenic and coculture conditions*.* Results unveiled the downregulation of key survival metabolites of *C. albicans* like myoinositol, C20 sphinganine 1-phosphate, farnesol, and gamma-undecalactone; therefore, explaining the antifungal potential of the endophyte crude extract [110]. The endophyte *Acremonium zeae* was isolated from maize plant and showed through paired culturing an antifungal potential against *A. flavus* and *F. verticillioides*, possibly due to a significant production of pyrrocidine antibiotic [111] (Fig. 9). In a study of *Nicotiana tabacum* L. (No. Y20210917) with its associated four endophytic fungal strains, *Penicillium janthinellum, Aspergillus fumigatus, Nigrospora sp. and Stagonosporopsis sp.,* the effect of the host media and coculturing was investigated and compared to the original axenic culture. Novel compounds as nigrolactone and multiplolide B were reported from the coculture of *Nigrospora *sp.* and Stagonosporopsis *sp. with antifungal activity against *Aspergillus fumigatus* down to MIC 2 μg/mL.; furthermore, the addition of the host extract to the fermentation media helped the production of AM6898A, asperfumol A, asperstone, and 4-epi-brefeldin C with no redundancy in pure PDB media. Even though 4β-acetoxyprobotryane-9β, 15α-diol was previously identified in *Botrytis cinere*a [112]*,* it was only obtained from the coculture of the two endophytic strains *Nigrospora *sp.* and Stagonosporopsis *sp. and absent in the tobacco host media, which manifested the power of coculture to inspire cryptic metabolites [19].

The coculture of the endophyte *Penicillium chrysogenum* with its host *Ziziphus jujuba* extract successfully directed the formation of cryptic rare metabolites as spiro-*β-*lactones and gem-dimethyl hydroxyl products, namely penicichrins A–C with potent activity towards *A. fumigatus* [107]. Red ginseng coculture with the endophyte *A. tubingensis* S1120 manifested a better antifungal effect than any of the plant or the monoculture endophytes against *A. tubingensis* with the production of aspertubin A and panaxytriol whose MIC values were about 8 μg/mL [113]. The butanolide derivative irperide was effective as antifungal against *A. fumigatus* with MIC value of 1 μg/mL [114].

Eremophilane sesquiterpenes and polyketide terpene hybrids from *Paraphaeosphaeria* endophytic species cultured with its host Gingko biloba fruit extracts revealed activity towards *Alternaria alternata* and *Beauveria bassiana*, yet only alternariol methyl ether showed promising antifungal effect against *A. fumigatus,* which was correlated to the chromen-6-one nucleus [115].

### D. Endophytic metabolites target fungal biofilms in selected pathogens.

**Fig. 10:**
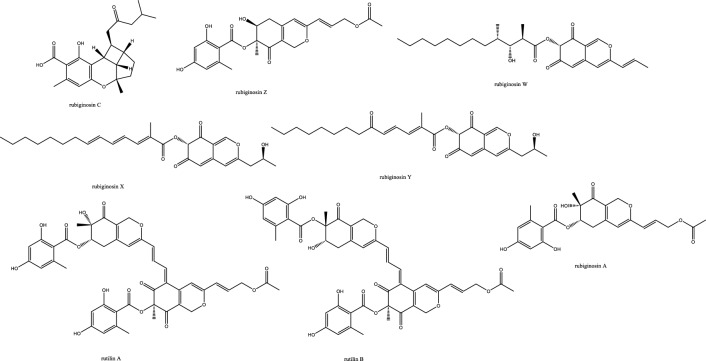
Endophytic compounds with potential biofilm inhibitory activity against selected priority pathogens

Biofilm formation is the culprit behind more than 80% of chronic and 60% of all microbial infections. Fungal biofilm is different from bacterial ones in composition and extracellular matrix, and while bacterial biofilms were subjected to better studies, those of fungi and yeast have only drawn attention recently [116]. In contrast to the free-living cells, fungi forming biofilms are more resistant to treatments and immune system defense mechanisms. *C. auris,* initially discovered in the external ear canal of a Japanese patient*,* was recalcitrant to multiple antifungal drugs like polyenes, azoles, and echinocandins; moreover, it is tolerant to high salt and high temperature conditions [117]. Zeng et al. reported rubiginosin C activity against both *C. albicans* and *C. auris* where it inhibited yeast to hyphae transformation and biofilm formation with non-significant cytotoxic effect on mammalian cells [118]. Rubiginosin C, isolated from the stromata of *Hypoxylon rubiginosum*, could be employed as an internal coat to medical devices in a pre-therapeutic application to protect polystyrene material from *C. albicans* or *C. auris* adhesion [119] (Fig. 10).

Rubiginosin C, derived from stromata of the ascomycete *Hypoxylon rubiginosum*, effectively inhibited the formation of biofilms, pseudohyphae, and hyphae in both *C. auris* and *C. albicans* without lethal effects. Crystal violet staining assays were utilized to assess the inhibition of biofilm formation, while complementary microscopic techniques, such as confocal laser scanning microscopy, scanning electron microscopy, and optical microscopy, were used to investigate the underlying mechanisms. Rubiginosin C is one of the few substances known to effectively target both biofilm formation and the yeast-to-hyphae transition of C. albicans and C. auris within a concentration range not affecting host cells, making it a promising candidate for therapeutic intervention in the future.

The lipophilicity and the long side chain of this azaphilone could contribute to its ease of access in biological membranes. *Alternaria tenuissima* OE7 endophytes isolated from *Ocimum tenuiflorum* L. leaves provided a bioactive ethyl acetate extract with a biofilm inhibitory activity against *C. albicans* at 1.0 mg/mL; moreover, the antifungal potential was evident towards several strains as *Candida albicans and C. tropicalis, Microsporum gypseum, A. parasiticus, Trichophyton rubrum, A. flavus, and A. fumigates.* The mode of action was examined by scanning electron microscopy and showed to be a fungicidal effect with hyphal and cellular destruction and a synergistic action when taken concomitantly with fluconazole [120].

### E. Future perspectives

Multi resistant fungal strains are growing more than any time before, and are considered a major health issue; particularly, to patients with invasive fungal infections affecting blood, brain, gut and lungs. Even worse our arsenal of antifungal drugs is limited, which makes drug discovery of novel antifungal a top health care priority. Recently, the WHO urged researchers to focus on hazardous fungi and yeasts and listed them as *A. fumigatus, C. albicans, C. auris and C. neoformans.* From the time 1980 to 2024 more than 300 compounds were isolated, identified and tested against these pathogens, yet few of them found their way to clinical trials and subsequently to the market. Our study realigned years of drug discovery against these four fungal and yeast strains and highlighted significant potent molecules to help their drug development process. We emphasize here the significance of endophytic polyketides where more than a third of the reported bioactive molecules belonged to this biogenic origin. Fungal biofilm inhibition is a promising research area in the following years and more studies are warranted in this realm since many molecules can be repurposed here in addition to novel compounds. Development of better culturing procedures can enhance fungal chemo diversity by applying modern OMICS techniques to unveil fungi dark matter. This includes but is not limited to coculturing endophytes either with other microorganisms or their host, which improves cultivability.

## Data Availability

Not applicable.
